# Comparison of the Self-Assembly and Conformations of Glucose- and Galactose-Based Glycopyranosides in Dilute Aqueous Solution

**DOI:** 10.1021/acs.langmuir.5c02346

**Published:** 2025-09-11

**Authors:** Ian W. Hamley, Anindyasundar Adak, Valeria Castelletto, Jani Seitsonen

**Affiliations:** † School of Chemistry, Food Biosciences and Pharmacy, 6816 University of Reading, Whiteknights, Reading RG6 6AD, U.K.; ‡ Nanomicroscopy Center, 174277 Aalto University, Puumiehenkuja 2, FIN-02150 Espoo, Finland

## Abstract

A series of glycolipids (glycopyranosides) was prepared by coupling glucosamine or galactosamine (at the C_2_ position) with myristic (tetradecanoic) or palmitic (hexadecanoic) acid. The conformation and self-assembly were examined in aqueous solutions containing 10% methanol. Circular dichroism and FTIR spectroscopy reveal notable differences in the chiral ordering and conformation comparing analogues with glucose and galactose “headgroups”. A glucose derivative (to palmitic acid) was also prepared with a different (C_1_-) substitution position of the lipid chain, and this was found to significantly influence chiral ordering and conformation. The self-assembly of the glycolipids was examined using cryogenic-transmission electron microscopy (cryo-TEM) and small-angle X-ray scattering (SAXS), which reveals lamellar structures, unilamellar for the glucose-based glycolipids, but multilamellar for the galactose-based analogues. Thus, the conformation and self-assembly of the molecules are very distinct, even though the glucose and galactose homologues have very similar structures, differing only in the orientation of a single hydroxyl group as epimers. These findings were rationalized with information from atomistic molecular dynamics (MD) simulations, which showed large differences in hydrogen-bonding density for glucose and galactose derivatives. The number of hydrogen bonds within interdigitated bilayers was much higher for the glucose variants, leading to stabilized unilamellar structures. The unexpectedly large differences in conformation and self-assembly of glycolipids bearing epimer monosaccharides may influence their properties and bioactivities.

## Graphical abstract

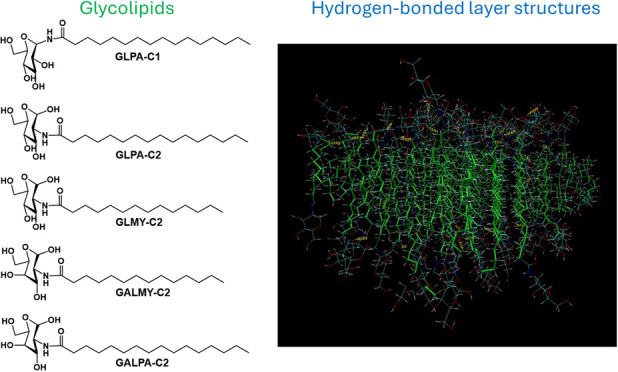

## Introduction

As their name suggests, glycolipids are molecules that contain lipid chains attached to glycosyl units, i.e., oligosaccharides (sugars). Glycolipids represent an important class of biomolecules present in the walls of different types of cells (e.g., bacterial, eukaryotic) and they modulate cell–cell interactions and influence other properties of the membrane. From a commercial and “green chemistry” perspective, glycolipids such as sophorolipids and rhamnolipids are currently attracting great interest as biosurfactants.^1,2^

The melt phase behavior of model glycolipids such as Guerbet glycolipids (with a branched alkyl chain) and others is remarkably rich, although smectic (lamellar) phases predominate. This has been extensively studied by several groups,^3–8^ and research on this topic has been reviewed.^9^ In one example, it has been demonstrated that stearyl glucosides show (nonhydrated) lamellar crystal (L_c_) structures at 25 °C, and fluid lamellar (L_α_) structures on heating, as shown by SAXS/WAXS, which also reveals that anomeric forms show different molecular packings in the lamellar phases.^10^ There have also been numerous studies on the self-assembly of glycolipids in concentrated aqueous solutions. Early studies on the lyotropic behavior (i.e., phase behavior in concentrated aqueous solution) of bacterial, plant, and mammalian glycolipids were reviewed.^11^ The structural properties of vesicles formed by several mono- and disaccharide glycolipids with C_10_–C_18_ alkyl chains were examined by SAXS/WAXS, which provides detailed information on the properties of the lamellae in gel and fluid phases, including layer spacings, hydration, areas per molecule, and others.^4^ Hashim and co-workers have investigated the lyotropic as well as the thermotropic liquid crystalline phases of Guerbet glycolipids, with lyotropic phases formed in concentrated solutions in excess water.^6,12^ The lyotropic polymorphism of long-chain alkyl glycopyranosides with a wide range of alkyl chain lengths and disaccharide headgroups has been examined, and a diverse range of lyotropic phases is observed in concentrated aqueous solutions.^5^ This group also examined the thermotropic and lyotropic behavior of glycopyranosides and galactopyranosides with monosaccharide headgroups and a range of lipid chains, including stearyl and oleyl. In concentrated aqueous solutions, they observed lamellar and other phases.^13^ Boyd and co-workers mapped the lyotropic phase behavior of alkyl α-D and β-d-glucosides and alkyl β-d-maltosides with octyl, decyl, or dodecyl chains. The phase diagrams showed micelle phases at low concentrations, lamellar phases at high concentrations, and hydrated crystals at low concentrations across a broad temperature range.^14^

There are few studies of the self-assembly behavior of glycolipids in dilute aqueous solutions. Shimizu and co-workers investigated a series of octadecyl (C_18_, stearyl) glucopyranosylamides with different saturated and unsaturated lipid chains, of which several were observed to form nanotubes in water (others showed amorphous or fibril morphologies).^15^ Baccile and co-workers report the pH-dependent self-assembly of β-d-glucose microbial glycolipids with saturated or monounsaturated lipid chains into vesicles, bilayers, or twisted fibrils.^16^ They also used SAXS and TEM to compare the self-assembled nanostructures of glucolipids bearing stearoyl or oleoyl lipid chains to a glucose or diglucose (sophorose) headgroup and found a similar diversity of morphologies.^17^ Lamellar hydrogels have also been observed for a stearoyl glucolipid under appropriate pH and concentration conditions.^18,19^

The self-assembly of α- and β-anomeric forms of disaccharide glycolipids has been compared using MD simulation and experimental methods. Small-angle scattering studies (SAXS and SANS) first revealed differences in shape and size of α- and β-anomers of n-dodecyl-d-maltoside undergoing self-assembly in water,^20^ later confirmed by MD simulations.^21^ The importance of hydrogen-bonding interactions between the sugar headgroups of the dodecyl-β-d-maltoside was later emphasized in the context of foam formation and monolayer formation at the air–water interface, based on atomistic MD and grazing-incidence X-ray diffraction studies.^22^ In a more recent example, SAXS and SANS revealed differences in the self-assembly of n-hexadecyl-d-maltopyranoside in its α- and β-anomeric forms in dilute aqueous solution. The α-form showed temperature- and concentration-dependent self-assembly (spherical micelles at low temperature and concentration), whereas semiflexible wormlike micelles were observed for the latter.^23^ Related work has been reviewed.^24^

We have previously compared the self-assembly in dilute aqueous solution of lipopolysaccharide (LPS) molecules, comparing two monodisperse lipid A derivatives based on simplified bacterial LPS structures to that of a native *E. coli* LPS using small-angle X-ray scattering (SAXS) and cryo-TEM.^25^ The *E. coli* LPS forms wormlike micelles, whereas the synthetic analogues bearing six lipid chains self-assemble into nanosheets or vesicles, depending on whether they contain four or two saccharide headgroups, respectively. These LPS molecules have lengthy polysaccharide chains, which have a strong effect on molecular packing and hence the observed nanostructure.^25^ In another remarkable example, the glycolipid oleoyl-β-d-glucose shows considerable polymorphism in dilute aqueous solution, including observed micelles, uni- and multilamellar lamellae, vesicles, complex coacervate structures, and fibers, depending on concentration, counterions, pH, and temperature.^26^

Here, we investigate and compare the self-assembly in a dilute aqueous solution of glycolipids bearing monosaccharide headgroups. We prepared several tetradecyl (C_14_, myristyl) or hexadecyl (C_16_, palmitoyl) glycolipids bearing either glucose (glucopyranose) or galactose (galactopyranose) saccharides. Glucose and galactose have the same chemical formula and are structural isomers (C_4_ epimers), but we show here that this leads to profound differences in self-assembled structures. For C_16_ glucopyranosylamides, we also compare the aggregation behavior of molecules with different attachment positions (C_1_ or C_2_ on the saccharide ring) of the lipid chain. Spectroscopic methods (FTIR and circular dichroism, CD) reveal differences in the hydrogen bonding and chirality, respectively, comparing glucose- and galactose-based homologues (and also comparing the effect of the C_1_ or C_2_ linking of the glucose group). The dilute solution nanostructures are determined through a combination of small-angle X-ray scattering (SAXS) and cryogenic-TEM (cryo-TEM), and distinct bilayer structures are observed for glucose or galactose-based glycolipids with monolayer or multilamellar structures, respectively. The experimental studies are complemented by atomistic molecular dynamics (MD) simulations, which provide information on the properties of modeled glycolipid bilayers, in particular, the extent of hydrogen bonding.

## Materials and Methods

### Materials

d-(+)-Glucosamine with the amine group at the C_1_ or C_2_ position was purchased from Sigma (UK). d-(+) Galactosamine was purchased from Biosynth (UK). Palmitic acid and myristic acid were purchased from TCI (UK). HBTU, dry DMF, and DIPEA were purchased from Thermo-Fisher (UK).

### Synthesis of Glycolipids

The palmitic acid or myristic acid (0.1953 mmol) was dissolved in dry DMF (2 mL) and stirred at 0 °C under an anhydrous nitrogen atmosphere for 5 min. Then, HBTU (0.3906 mmol) and DIPEA (1.1718 mmol) in 3 mL of dry DMF were added dropwise to this solution. The resulting mixture was left stirring for another 10 min. Then, d-(+)-glucosamine/galactosamine (0.2343 mmol) was added to the reaction mixture and kept under stirring conditions at room temperature. After 24 h, DMF was evaporated by rotary evaporation, and column chromatography (dichloromethane/methanol = 5:1) was performed to obtain the pure product. The yields were 32.2% for GLPA-C1, 31.7% for GLPA-C2, 33.52% for GLMY-C2, 30.41% for GALMY-C2, and 32.94% for GALPA-C2.

### Dissolution of Glycolipids

The glycolipids were not fully soluble in aqueous solution but could be dissolved in 90% water:10% methanol solutions. These conditions were used for all of the subsequent experiments.

### Circular Dichroism (CD) Spectroscopy

Far-UV CD spectra were collected using a Chirascan spectropolarimeter (Applied Photophysics, Leatherhead, UK) equipped with a thermal controller. Spectra were recorded from 180 to 400 nm. Samples were mounted in a quartz cell with detachable windows with a 0.01 nm path length. The CD signal from the samples was corrected by water background subtraction. The CD signal was smoothed using Chirascan Software for data analysis. The residual was chosen to oscillate around the average to avoid artifacts in the smoothed curve. CD data, measured in mdeg, was normalized to molar ellipticity using the molar concentration of the sample and the cell path length.

### Fourier-Transform Infrared (FTIR) Spectroscopy

FTIR spectra were obtained using a Thermo-Scientific Nicolet iS5 instrument with a DTGS detector. The solution was placed in a Specac Pearl liquid cell with CaF_2_ plates. For each sample, a total of 128 scans were recorded over the range of 900–4000 cm^–1^.

### Cryogenic-TEM (Cryo-TEM)

Imaging was carried out using a field emission cryoelectron microscope (JEOL JEM-3200FSC), operating at 200 kV. Images were taken in bright field mode and using zero-loss energy filtering (omega type) with a slit width of 20 eV. Micrographs were recorded using a Gatan Ultrascan 4000 CCD camera. The specimen temperature was maintained at −187 °C during the imaging. Vitrified specimens were prepared using an automated FEI Vitrobot device using Quantifoil 3.5/1 holey carbon copper grids with a hole size of 3.5 μm. Just prior to use, grids were plasma-cleaned using a Gatan Solarus 9500 plasma cleaner and then transferred into the environmental chamber of an FEI Vitrobot at room temperature and 100% humidity. Thereafter, 3 μL of sample solution was applied on the grid, blotted twice for 5 s, and then vitrified in a 1/1 mixture of liquid ethane and propane at a temperature of −180 °C. The grids with vitrified sample solution were maintained at the liquid nitrogen temperature and then cryo-transferred to the microscope.

### Small-Angle X-ray Scattering (SAXS)

SAXS experiments were performed on beamline B21 ^27^ at Diamond (Didcot, UK). The sample solutions were loaded into the 96-well plate of an EMBL BioSAXS robot and then injected via an automated sample exchanger into a quartz capillary (1.8 mm internal diameter) in the X-ray beam. The quartz capillary was enclosed in a vacuum chamber to avoid parasitic scattering. After the sample was injected into the capillary and reached the X-ray beam, the flow was stopped during the SAXS data acquisition. Beamline B21 operates with a fixed camera length (3.9m) and fixed energy (12.4 keV). The images were captured by using a PILATUS 2 M detector. Data processing was performed by using dedicated beamline software ScÅtter.

### Molecular Dynamics Simulations

Molecular dynamics simulations were performed using Gromacs^28^ (versions 2024.4, 2023.2, or Ubuntu-2020.1–1). Molecules of each of the five glycolipids were packed using Packmol^29^ into interdigitated bilayers of 80 molecules. The influence of the effect of the presence or absence of hydrogen atoms was also explored in some simulations. Each set of glycolipid simulation parameters was generated using the Glycan Reader and Modeler^30,31^ of CHARMM-GUI.^32,33^ Simulations were performed using the CHARMM36 force field.^34,35^ The initial bilayers were placed into simulation boxes (cubes) of length 8 nm, and the systems were solvated using TIP3P water. After energy minimization and 100 ps relaxation stages in the NVT ensemble, the final simulations were carried out in the NPT ensemble using a leapfrog integrator with steps of 1 fs up to 1 or 4 ns, depending on the equilibration of the system. The temperature was maintained at 303.13 K using the velocity-rescale (modified Berendsen) thermostat^36^ with a coupling constant of 10 steps. The pressure was maintained at 1 bar using the Parinello–Rahman barostat,^37^ and periodic boundary conditions were applied in all three dimensions. The Particle Mesh Ewald scheme^38,39^ was used for long-range electrostatics. Bonds were constrained using the LINCS algorithm,^40^ and the Verlet cutoff scheme^41^ was used. Coulomb and van der Waals cutoffs were 1.0 nm.

## Results and Discussion

The glycolipids were prepared by a simple coupling reaction of palmitic (hexadecenoic) acid or myristic (tetradecanoic) acid to activate glucosamine or galactosamine to produce the compounds shown in Figure 1a. Two variant conjugates of palmitic acid with glucosamine were prepared, with substitution at the C_1_ or C_2_ position (Figure 1b), producing conjugates termed GLPA-C1 and GLPA-C2, respectively. The synthesized compounds were purified by column chromatography and characterized by using electrospray ionization mass spectroscopy and ^1^H and ^13^C NMR spectroscopy. The characterization data for the five compounds prepared are presented in SI, Figure S1–S20, which confirm the expected structures and high purity. When a lipid is attached at C_1_, glucose becomes a glycoside, which does not undergo mutarotation and remains fixed in its α- or β-configuration.^42^ If the lipid is attached at any other position (e.g., C_2_–C_6_), then the anomeric carbon (C_1_) is free to open and close (mutarotate). There will be an equilibrium mixture of α- and β-anomers in solution and not two distinct, isolatable compounds. Studies suggest that the mutarotation of glucose involves reversible conversion between α- and β-anomers through mechanisms that require a free anomeric position. Attaching a large lipid chain to the C_1_ position would prevent mutarotation by blocking this process. We performed an NMR study of GLPA-C1 with and without water and found that there is no extra anomeric peak; only a slight shift of the C_1_ hydrogen peak is observed (SI Figure S21), with a deshielding effect, which is also observed for the amide proton, although the peak position of the aliphatic lipid chain remains the same. This suggests possible hydrogen bonding during the self-assembly process.

**Figure 1:**
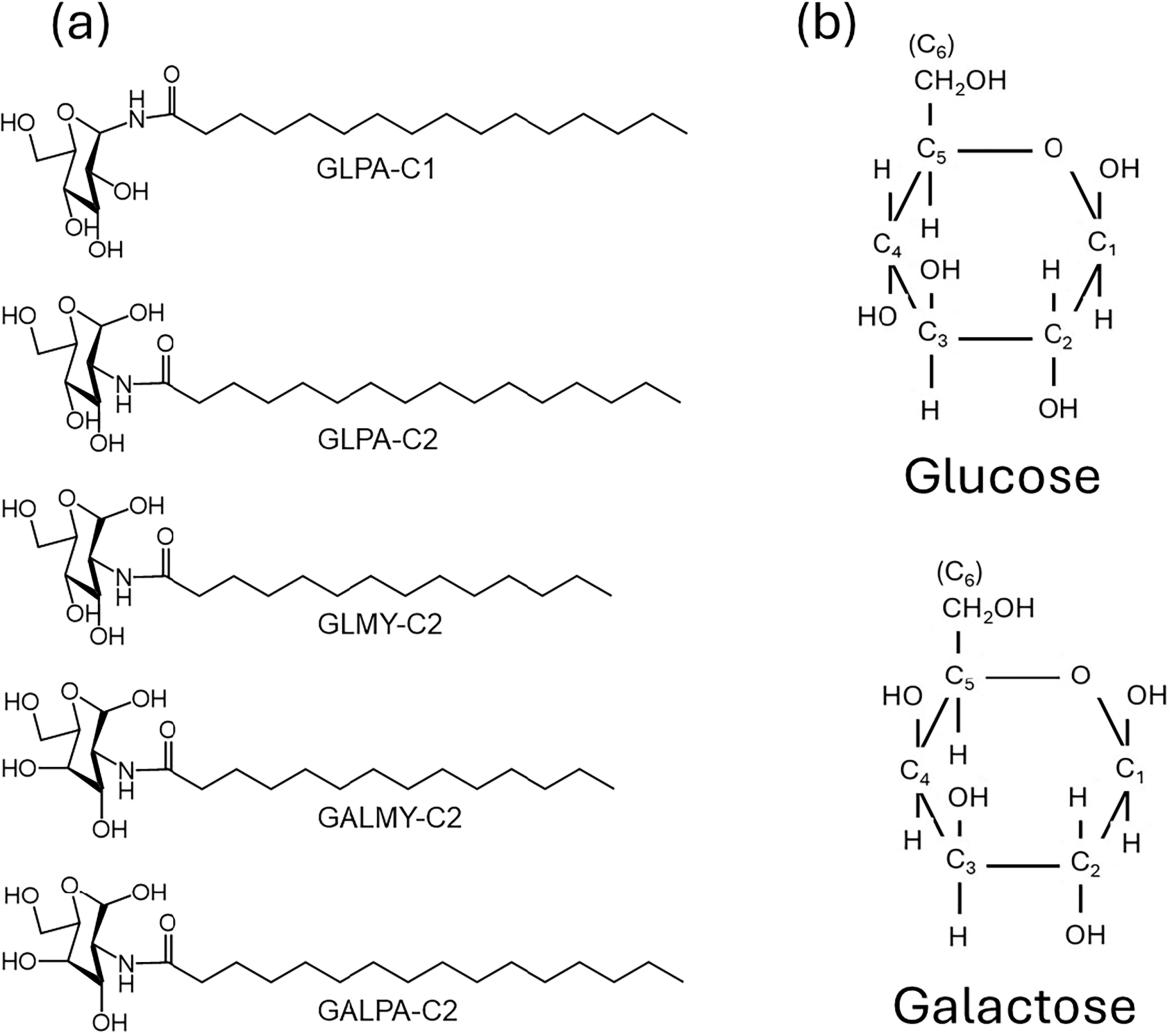
(a) Molecular structures of glycolipids studied. (b) Cartoon of glucose and galactose rings (N.B. in reality, the rings are not flat and the bond and torsional angles differ from the idealization).

Glucose and galactose have very closely related structures (epimers), as shown in Figure 1b. The only difference is the orientation of the hydroxyl group at C_4_. Nevertheless, as described in the following, we found that this leads to significant differences in conformation of glycolipids bearing these moieties, as well as in the self-assembled nanostructures in aqueous solution.

The conformation of the monosaccharide “headgroups” in the glycolipids was probed via CD spectroscopy. The spectra are shown in Figure 2 and show distinct differences depending on the type of saccharide, as well as the lipid and its linking position. One family of spectra is obtained for GLMY-C2 and GLPA-C2 with C_2_-attached glucose. The spectra are characterized by a negative peak centered at 206 nm. Negative CD peaks were previously reported for glucopyranosylamide lipids with a main peak at 230 nm for solutions in water, where the glycolipids formed nanotubes.^15^ The negative Cotton effect in the CD spectra is due to a left-rotation center. The CD spectra in Figure 2 for the two galactose-based glycolipids are very distinct from those for the glucose-based compounds and show positive Cotton effects (with a positive peak with a maximum at 213 nm for GALPA-C2 or 217 nm for GALMY-C2). This differs from the CD spectrum with a negative band centered at 220 nm observed for fibril-forming N-oleoyl β-d-galactopyranosylamine in water^43^ or a hydrolyzed cellobioselipid mixture^16^ (forming chiral helical ribbon structures), although in the same paper, featureless CD spectra were reported for oleoyl- and stearoyl-glucolipids.^16^ The CD spectrum for GLPA-C1, with a different lipid chain attachment point (C_1_) compared to the other two glucose-based glycolipids studied, shows a further distinct profile, with two positive maxima at 194 and 211 nm (Figure 2).

**Figure 2:**
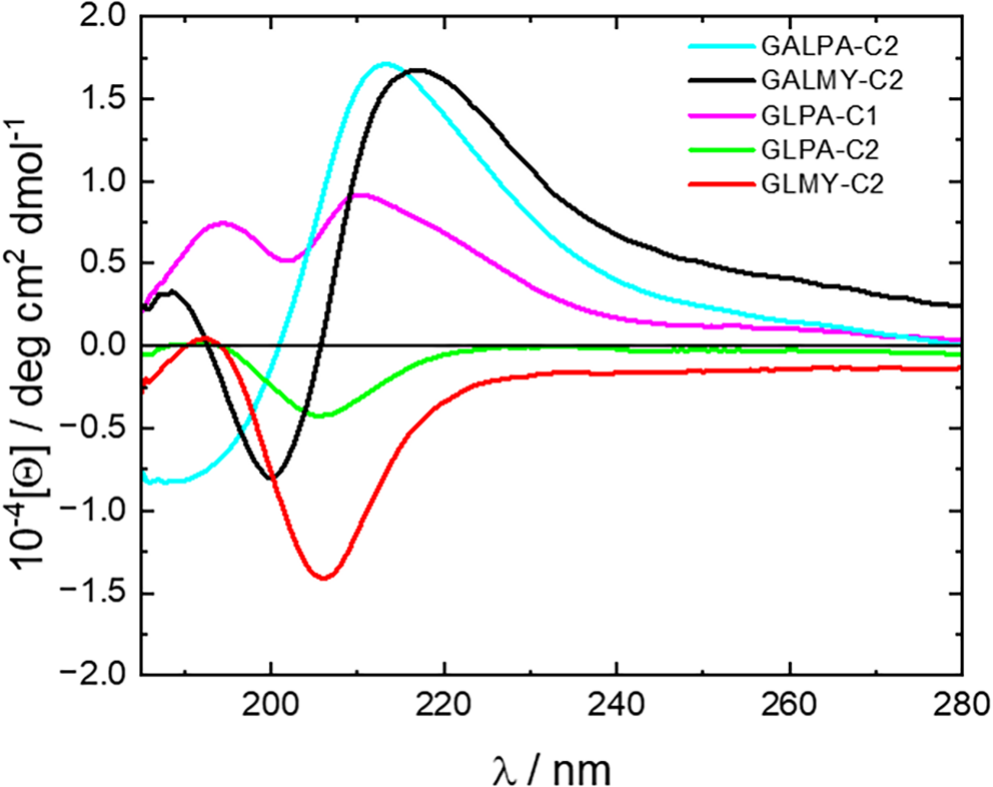
CD spectra for 0.1 wt % solutions in 10 wt % methanol/90 wt % water.

CD spectroscopy was complemented by FTIR spectroscopy, which also shows distinct features for the different classes of glycolipids. The FTIR spectra are shown in Figure 3. Peaks in the range 1460–1470 cm^–1^ are due to methylene scissoring δ­(CH_2_) vibrations.^44^ There are subtle differences in the features for the two galactose-based lipids with a peak at 1462 cm^–1^ (and smaller peaks near 1473 cm^–1^) compared to the glucose-based lipids with peaks at 1467–1469 cm^–1^, pointing to differences in the lability of methylene groups in the two sugars. The region of the spectra around 1550 cm^–1^ due to N–H vibrations^45^ also shows differences, although with no notable trends. There are very marked differences in the amide I′ region,^44^ with sharp peaks at 1606 cm^–1^ for GALPA-C2 and GALMY-C2 or broad peaks around 1631–1634 cm^–1^ for GLPA-C1 and GLPA-C2 (with the latter peak broader with an additional feature at 1639 cm^–1^). The spectrum for GLMY-C2 in contrast shows no peak in this range. These features suggest differences mainly in C=O stretch deformations with contributions from C–N and N–H deformation modes when comparing the two types of glycolipids. The amide I′ peaks for the glucose-based glycolipids are blue-shifted (hypsochromic), possibly due to strengthened intermolecular C=O···H–N hydrogen bonds. This will be discussed further in view of the MD results below.

**Figure 3:**
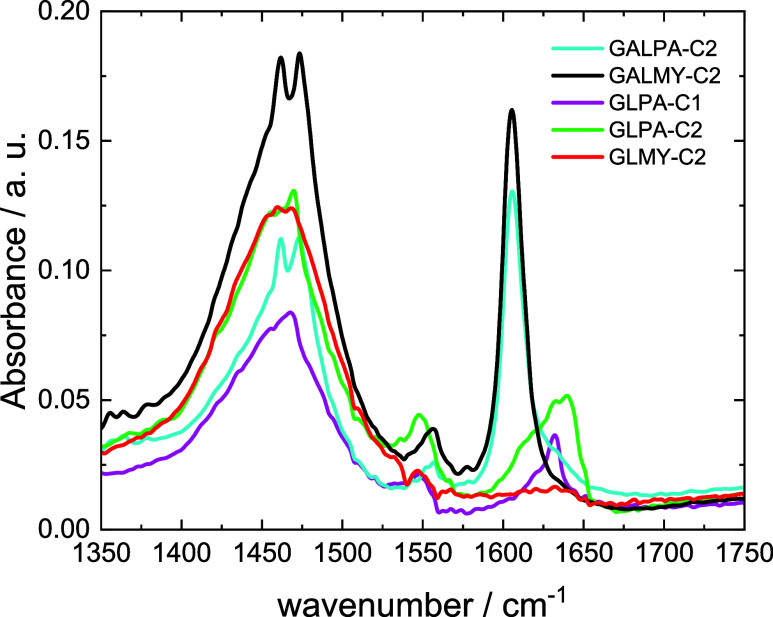
FTIR spectra for 0.1 wt % solutions in 10 wt % methanol/90 wt % water.

To summarize the spectroscopic studies, the CD and FTIR spectra point to significant differences in chirality and hydrogen bonding between the glucose- and galactose-based glycolipids, with GLPA-C1 additionally showing distinct conformational properties due to the different positions of lipid chain anchoring. The differences in hydrogen bonding and chirality are related below to the formation of distinct superstructures, as revealed by cryo-TEM, SAXS, and atomistic molecular dynamics simulations.

The self-assembly of the glycolipids was investigated by cryo-TEM imaging combined with SAXS. Figure 4 shows representative cryo-TEM images, and qualitative differences are apparent when comparing the three glucose-based glycolipids (images in the top row) with the galactose-based ones (images in the bottom row). Additional cryo-TEM images are included in SI, Figure S22. The GL-based glycolipids form irregular thin nanosheet-like structures and aggregates around the edges of the TEM grid holes. In contrast, for the galactose-based glycolipids GALMY-C2 and GALPA-C2, well-defined plate-like sheet structures with, in many cases, polyhedral edges are apparent. The cryo-TEM suggests that the GL-based glycolipids may form mainly unilamellar nanosheet structures, whereas the GAL-containing ones form multilayer sheets. In a few regions, periodic fringes from multilayer structures could be observed for the GL-based glycolipid GLPA-C2, as shown in SI, Figure S23. The periodic spacing is 4.9 nm, which is in reasonable agreement with the SAXS data discussed below. The difference in lamellar stacking of GL- and GAL-glycolipids is supported by SAXS, based on the data shown in Figure 5. The data for all samples is characterized by an intensity scaling at low wavenumber *q*, *I* ∼ *q* ^–2^, consistent with the formation of layered structures.^46^ However, the data also reveal notable differences comparing GAL and GL-glycolipids. Sharp Bragg peaks are present in the data for the two GAL-glycolipids but not the GL-based ones for which broader peaks are observed (this is further evidenced by the expanded scale; linear *q* scale representation of the data in SI, Figure S24a). The peaks for the GL-glycolipids can be interpreted as broad Bragg peaks; indeed, fitting of the SAXS data suggests that they relate to structures with approximately *N* = 5–6 layer repeats, as shown by the representative fit in SI, Figure S24b. The Bragg peak for GALPA-C2 (with a C_16_ palmitoyl lipid chain) is at *q* = 0.153 Å^–1^, corresponding to a layer spacing *d* = 41.2 Å, and for GALMY-C2, the peak is at *q* = 0.167 Å^–1^, corresponding to a shorter layer spacing *d* = 37.6 Å, consistent with the shorter lipid chain in the C_14_ (myristoyl) conjugate. There is a higher-order peak centered at *q* = 0.18 Å^–1^ for GALPA-C2 (Figure 4 and SI, Figure S24), which is significantly broader than the first-order peak and is thus assigned to a coexisting phase with a lower number of lamellar repeats. Considering the molecular lengths (length of the C_16_ chain taken as 18 Å, nm, approximately +5 Å for the sugar group), these layer spacings are consistent with bilayer packings of the molecules, with limited interdigitation. Broad maxima at low *q* values in the data for GLMY-C2 and GLPA-C2 are ascribed to structure factor features. The SAXS data for GLPA-C2 and GLMY-C2 shows a reproducible broad peak near *q* = 0.01 A^–1^, which may be due to correlations between lamellae although fitting of the data using a combination of a bilayer form factor^47^ and a lamellar structure factor^48^ (SI, Figure S24b), used by our group to fit SAXS data from lipopeptide lamellar structures,^49,50^ also suggests that it can arise from the form factor features of bilayer structures.

**Figure 4:**
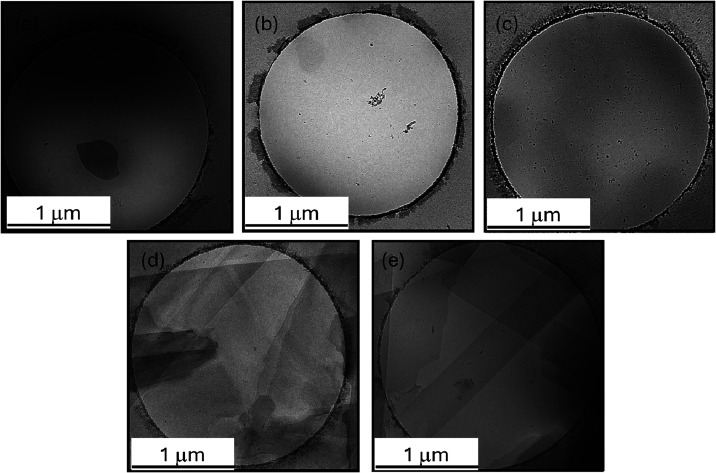
Cryo-TEM images for 0.1 wt % solutions in 10 wt % methanol/90 wt % water. (a) GLPA-C1, (b) GLPA-C2, (c) GLMY-C2, (d) GALMY-C2, and (e) GALPA-C2.

**Figure 5:**
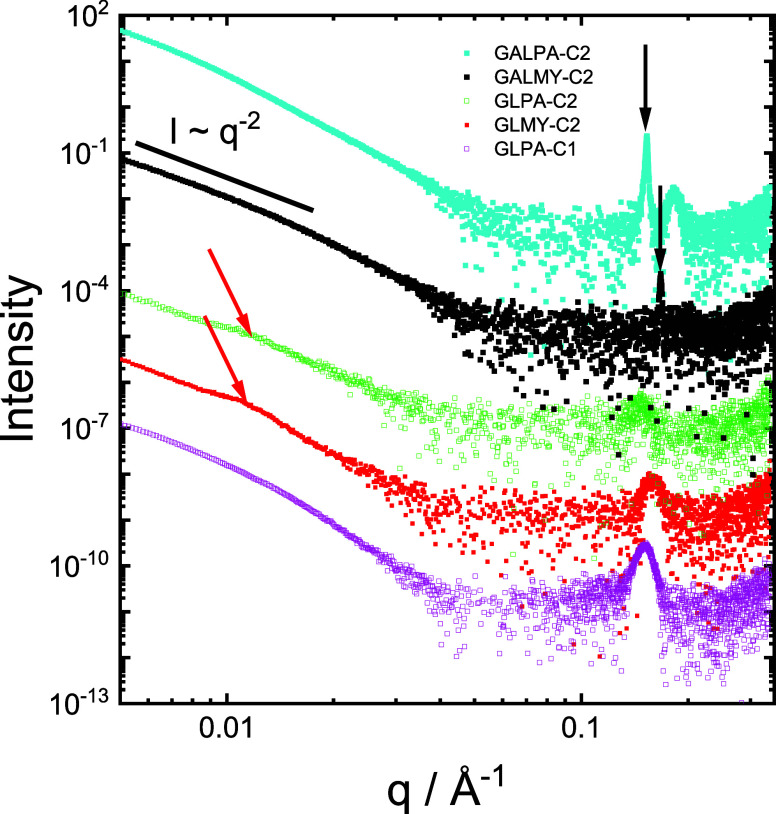
SAXS data for 0.1 wt % solutions in 10 wt % methanol/90 wt % water, as indicated. Bragg peaks are highlighted with black arrows, and broad, weak structure factor features with red arrows. Data are offset for ease of visualization.

There appears to be an inter-relationship between conformational properties of GAL-glycolipids revealed by CD and FTIR and the formation of a multilamellar structure for these two compounds, as revealed by cryo-TEM. We turned to atomistic molecular (MD) dynamics simulations to further examine this. MD was performed on 80-molecule bilayer structures (with interdigitated lipid chains and glycol groups at each surface, consistent with the SAXS data), which were found to be stable under the simulation conditions employed. Figure 6 shows, as an example, projections of the structure from the final frame of the MD run for GLPA-C2. During the course of the simulation, the development of pseudohexagonal in-plane order of the packed glycolipid molecule was noted. Images showing this in-plane order for all five glycolipids are provided in SI, Figure S25. That the simulation reached equilibrium was confirmed by monitoring the RMSD (root-mean-square deviation of positions) and Rg (radius of gyration) of the system, as well as by analyzing solvent-accessible surface area properties (SASA, Δ*G*_solv_, volume, density), as shown in SI, Figure S26. The decrease in SASA values can be used to determine an aggregation propensity (AP) defined as the ratio of initial/final SASA.^51^ The AP values from the simulations are 1.10 for GLPA-C1, 1.16 for GLPA-C2, 1.04 for GLMY-C2, 1.65 for GALMY-C2, and 1.23 for GALPA-C2. These values indicate a notably higher aggregation potential for the GAL-based glycolipids (especially GALMY-C2), consistent with the findings of extensive multilamellar structures from cryo-TEM and SAXS.

**Figure 6:**
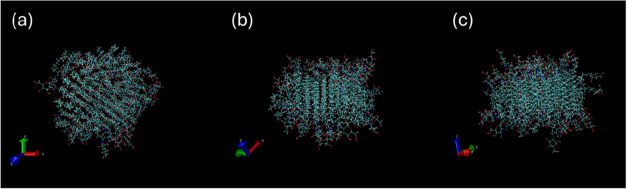
Representative images of the bilayer structure from MD simulations: projections of the final frame (*t* = 1 ns) for GLPA-C2. (a) View along the layer normal. (b, c) Side views of the layer.

To compare one GL-based molecule with the GAL analogue, a more detailed analysis of conformation was undertaken for GLPA-C2 and GALPA-C2. Among other quantities examined (including interatomic distances and hydrogen bond numbers, discussed below), to probe alignment of molecular conformations, we examined the orientation of the lipid chain with respect to vectors in the saccharide head (including the C_4_ stereocenter and C_1_ anomeric center),^8^ as shown in SI, Figure S27, as well as angles within the pyranose group. The angles of lipid chains with respect to vectors across the saccharide headgroup (C_4_–C_1_) or (C_5_–C_2_) are very similar for both GLPA-C2 and GALPA-C2 (SI, Figure S28), and this suggests that the packing of the molecules as reflected by these vectors is not substantially different, although the angle between the lipid chain and (C_4_–C_1_) is slightly lower for GLPA-C2. The (C_4_–C_1_)–(C_5_–C_2_) angles are also the same for the two sugar isomers. The angles associated with the orientation of the hydroxyl group on the C_4_ stereocenter with respect to C_6_ are different (SI, Figure S28), as expected based on the expected down- or up-alignment of the C_4_ hydroxyl with respect to the ring, as sketched in Figure 1b, which is in fact the distinction between β-d-glucose and β-d-galactose.

Since intermolecular vector analysis did not reveal significant differences between the GL and GAL-based glycolipid assembly structures, the number of H-bonds within the simulated systems was also analyzed. A significantly higher number of H-bonds was observed for each glucose molecule compared to its galactose analogue, as exemplified by the data in Figure 7 comparing GLMY-C2 and GALMY-C2 and SI, Figure S29 comparing GLPA-C2 and GALPA-C2. The enhancement of hydrogen bonding within the single-layer simulations for the glucose-based glycolipids may stabilize the formation of monolayers for these glycolipids, whereas lower intralayer hydrogen bonding (or competitive interactions, e.g., solvation of −OH groups) may reduce this tendency and favor multilamellar structures for the galactose-based glycolipids.

**Figure 7:**
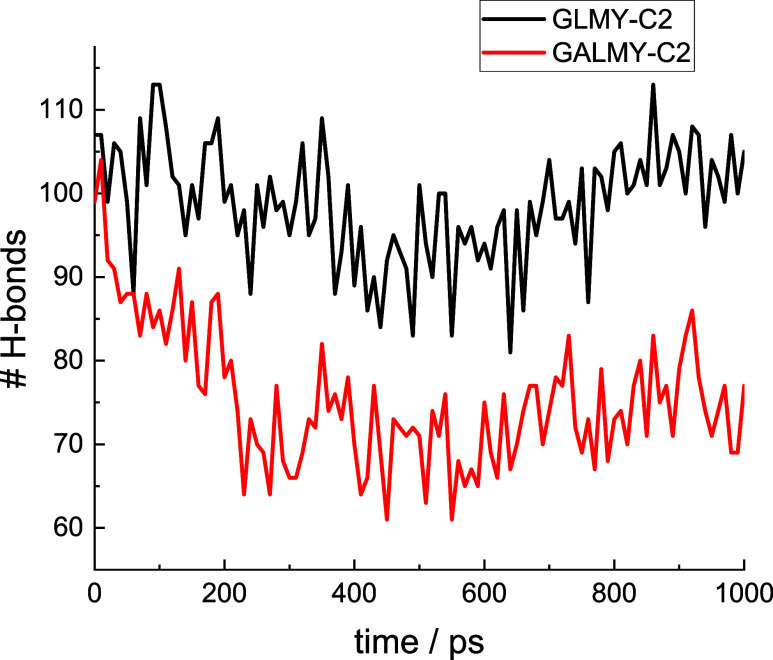
Time evolution of the numbers of hydrogen bonds (within the whole system) for GLMY-C2 and GALMY-C2.

## Conclusions

In summary, a series of glucose- and galactose-based glycolipids have been synthesized. Circular dichroism and FTIR spectroscopy reveal significant conformational differences comparing GAL-based glycolipids with their GL-based analogues, and the position of substitution also influences this, comparing the conformation of GLPA-C1 and GLPA-C2. The most noticeable difference from FTIR is the large, sharp peak due to amide I deformation modes observed for GALPA-C2 and GALMY-C2; this is replaced by a broader, weaker peak at a higher wavenumber for the GL-based analogues. This indicates significant differences in the extent of hydrogen bonding, which was further investigated using atomistic molecular dynamics simulations. The chirality revealed by CD spectroscopy is different, comparing the two classes of molecules, with a further difference in the sign (as well as the shape of the spectrum) of the CD signal (Cotton effects) comparing the C_1_-attached GLPA-C1 to the C_2_-attached homologue GLPA-C2. The different CD spectra are due to local electronic structure around the stereocenter, which could be examined with molecular quantum mechanical methods. The FTIR spectra also depend very sensitively on the deformation modes of different bonds, which are affected by the local environment, e.g., local hydration, although this is challenging to model.

The differences in conformation and especially intermolecular hydrogen bonding give rise to distinct self-assembled structures comparing the GAL-C2 and GL-C2 pairs of glycolipids. Cryo-TEM imaging and SAXS studies reveal unexpected differences in the nanostructure arising from the packing of glucose versus galactose homologous glycolipids. The GL-based molecules form irregular monolayers, whereas the GAL-glycolipids form multilamellar crystallite-like structures. Atomistic MD indicates a significantly higher number of hydrogen bonds for the GL molecules in a simulated leaflet, which may stabilize the observed monolayer structures. As mentioned above, the importance of hydrogen bonding of sugar units in glycolipids has been revealed in MD and other studies.^22^ This is also shown through studies on the self-assembly of bolaamphiphiles bearing terminal 1-glucosamide groups; a hydrogen-bonded layered crystal structure is observed for some molecules (depending on the central alkyl chain)^52,53^ Similarly, the crystal structure of 1-galactosamide bolaamphiphiles shows extensive H-bond networks of the galactose hydroxyl groups.^54^ We have uncovered significant differences in conformational and self-assembly behavior comparing glucose and galactose homologue glycolipids that may be expected to influence their properties, such as bioactivities, especially relevant to the control of cell membrane interactions.

## Supplementary Material



## Notes

Molecular characterization data (ESI-MS and ^1^H and ^13^C NMR spectra), additional cryo-TEM images, additional plot of SAXS data, snapshots from MD simulations, SASA- and angle-distribution related properties from MD simulations and numbers of hydrogen bonds for GLPA-C2 and GALPA-C2, and a Table of SAXS fit parameters (PDF)
