# Supplementary material for: Comparison of the Self-Assembly and Conformations of Glucose- and Galactose-Based Glycopyranosides in Dilute Aqueous Solution
Source: Langmuir. 2025 Sep 11;41(37):25221–9. doi: 10.1021/acs.langmuir.5c02346 (PMC12461907; doi:10.1021/acs.langmuir.5c02346)
Supplement: Supplementary file 1 [file la5c02346_si_001.pdf]

Supporting Information

Comparison of the Self-Assembly and Conformations of Glucose- and Galactose-Based Glycopyranosides in Dilute Aqueous Solution

Ian W. Hamley,[1,*] Anindyasundar Adak, [1] Valeria Castelletto,[1] Jani Seitsonen,[2]

[1] School of Chemistry, Food Biosciences and Pharmacy, University of Reading, Whiteknights, Reading RG6 6AD, U.K.

[2] Nanomicroscopy Center, Aalto University, Puumiehenkuja 2, FIN-02150 Espoo, Finland

* Author for correspondence: I.W.Hamley@reading.ac.uk

Number of pages: 28

Number of figures: 29

Number of tables: 1

Table of contents:

S-3… **SI Fig. S1.** Palmitic acid conjugated at C1 position of Glucose **(GLPA-C1)**

S-3… **SI Fig. S2.** Mass spectrometry data of GLPA-C1**.**

S-4… **SI Fig. S3.** ^{1}H NMR data of GLPA-C1**.**

S-5… **SI Fig. S4.** ^{13}C NMR data of GLPA-C1**.**

S-6… **SI Fig. S5.** Palmitic acid conjugated at the C2 position of Glucose **(GLPA-C2)**

S-6… **SI Fig. S6.** Mass spectrometry data of GLPA-C2**.**

S-7… **SI Fig. S7.** ^{1}H NMR data of GLPA-C2**.**

S-8… **SI Fig. S8.** ^{13}C NMR data of GLPA-C2**.**

S-9… **SI Fig. S9.** Myristic acid conjugated at the C2 position of Glucose **(GLMY-C2)**.

S-9… **SI Fig. S10.** Mass spectrometry data of GLMY-C2**.**

S-10… **SI Fig. S11.** ^{1}H NMR data of GLMY-C2.

S-11… **SI Fig. S12.** ^{13}C NMR data of GLMY-C2.

S-12… **SI Fig. S13.** Myristic acid conjugated at the C2 position of Galactose **(GALMY-C2)**.

S-12… **SI Fig. S14.** Mass spectrometry data of GALMY-C2.

S-13… **SI Fig. S15.** ^{1}H NMR data of GALMY-C2.

S-14… **SI Fig. S16.** ^{13}C NMR data of GALMY-C2.

S-15… **SI Fig. S17.** Palmitic acid conjugated at the C2 position of Galactose **(GALPA-C2)**.

S-15… **SI Fig. S18.** Mass spectrometry data of GALPA-C2.

S-16… **SI Fig. S19.** ^{1}H NMR data of GALPA-C2.

S-17… **SI Fig. S20.** ^{13}C NMR data of GALPA-C2.

S-18… **SI Fig. S21**. ^{1}H NMR spectra of GLPA-C1 in DMSO-d_6 and water.

S-19… **SI Fig. S22.** (a) Additional cryo-TEM images for 0.1 wt% solutions in 10 wt% methanol/90 wt% water, (b) Enhanced contrast images.

S-20… **SI Fig.S23**. Example of region of cryo-TEM image from GLPA-C2 showing fringes from small multilamellar stack along with cross-section analysis to estimate periodicity, 4.8 nm.

S-21… **SI Fig. S24.** (a) SAXS data (same data as Fig.5) on expanded linear *q* scale. Data are offset for ease of visualization, (b) Fit of data for GLPA-C2 (double log scale, fit parameters in SI Table S1). For ease of visualization, only every 3rd data point is shown.

S-22… **SI Fig. S25.** Images of in-plane ordering from final frames of MD simulations.

S-22… **SI Fig. S26.** SASA-related properties from MD simulations

S-23… **SI Fig. S26.** SASA-related properties from MD simulations

S-24… **SI Fig. S26.** SASA-related properties from MD simulations

S-25… **SI Fig.S27**. Molecular structures of GLPAC2 and GALPAC2 and definitions of vectors for MD angle distribution studies.

S-26… **SI Fig.S28**. Angles associated with packing of lipid chains/headgroups and vectors within sugar rings from MD simulations for (a) GLPA-C2, (b) GALPA-C2. See Fig.1b and SI Fig.S25 for atom labelling.

S-27… **SI Fig.S29**. Time evolution of numbers of hydrogen bonds (within the whole system) for GLPA-C2 and GALPA-C2.

S-28… **Table S1.** Parameters extracted from the fitting of the SAXS data in Fig.5 (and SI Fig.S22b) for GLPA-C2.

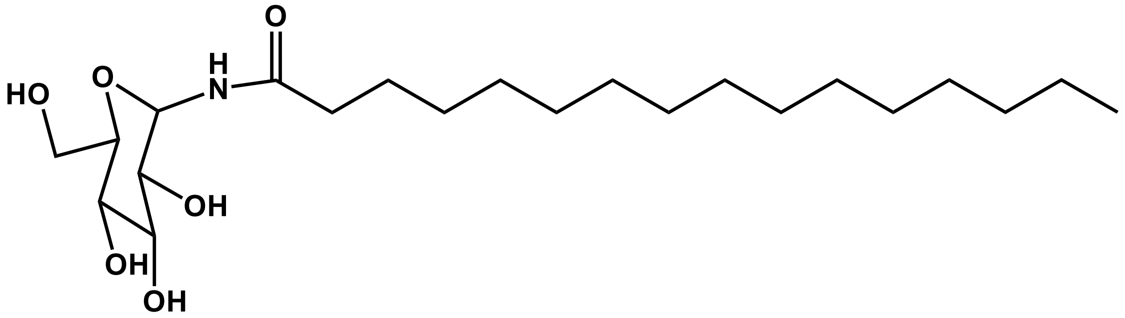

SI Fig. S1. Palmitic acid conjugated at C1 position of Glucose **(GLPA-C1)**

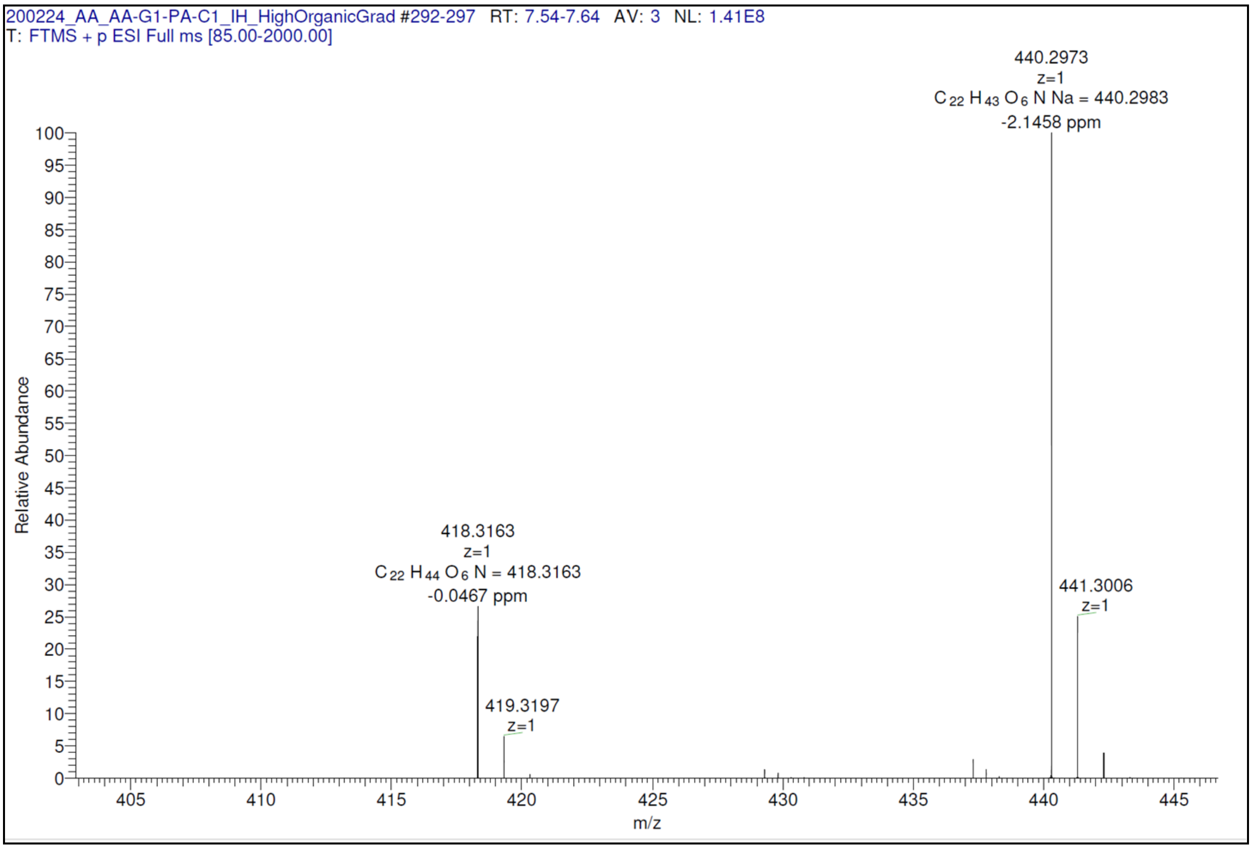

SI Fig. S2. Mass spectrometry data of GLPA-C1. $M_{(calculated)} = 417.59$ g mol^{-1}, $M_{(observed)}$ = 418.31 g mol^{-1} (M+H), and 440.28 g mol^{-1} (M+Na).

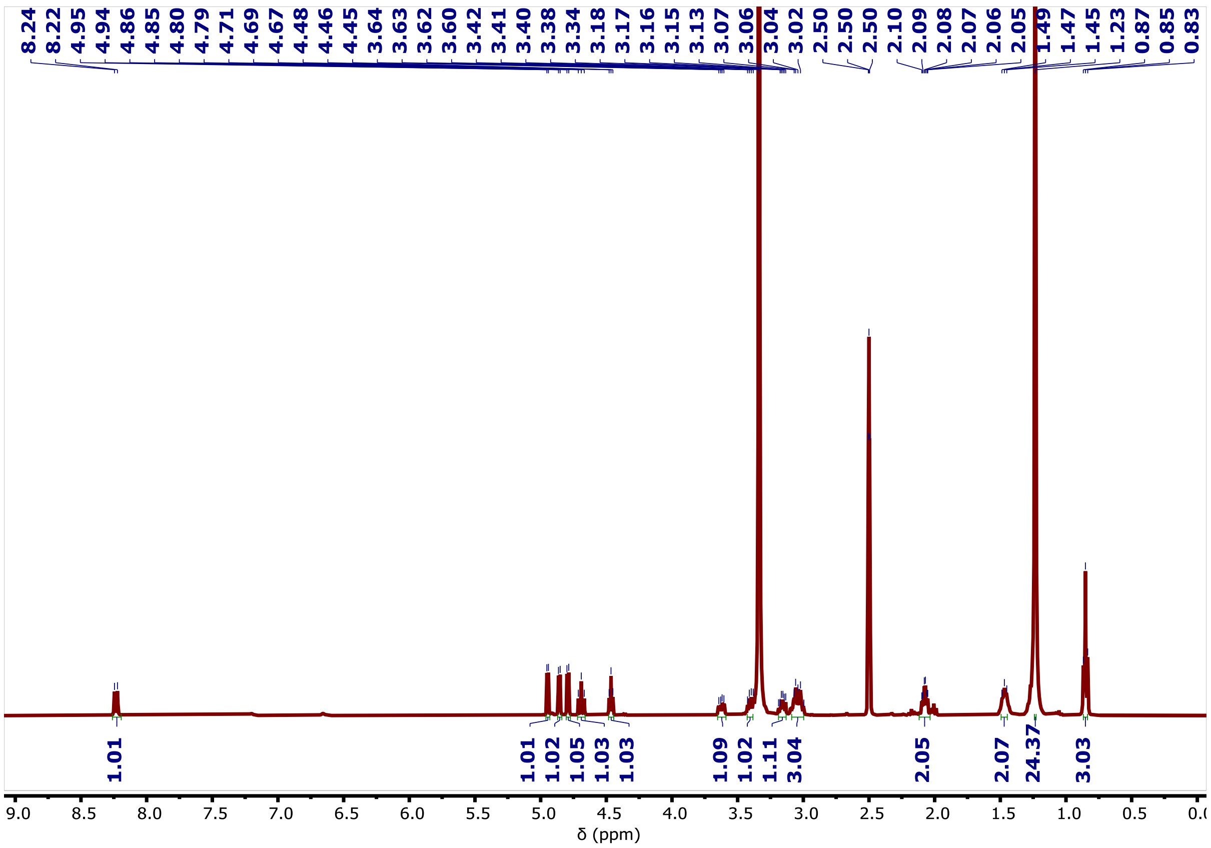

SI Fig. S3. ^{1}H NMR data of GLPA-C1.

^{1}H NMR (400 MHz, DMSO-d_6) δ 8.23 (d, J = 9.1 Hz, 1H), 4.95 (d, J = 4.7 Hz, 1H), 4.86 (d, J = 5.1 Hz, 1H), 4.79 (d, J = 5.6 Hz, 1H), 4.69 (t, J = 9.1 Hz, 1H), 4.46 (t, J = 5.8 Hz, 1H), 3.62 (dd, J = 9.8, 5.6 Hz, 1H), 3.43 – 3.38 (m, 1H), 3.16 (td, J = 8.6, 4.7 Hz, 1H), 3.05 (dd, J = 13.2, 6.1 Hz, 3H), 2.07 (td, J = 7.3, 3.0 Hz, 2H), 1.46 (d, J = 7.3 Hz, 2H), 1.23 (s, 24H), 0.85 (s, 3H).

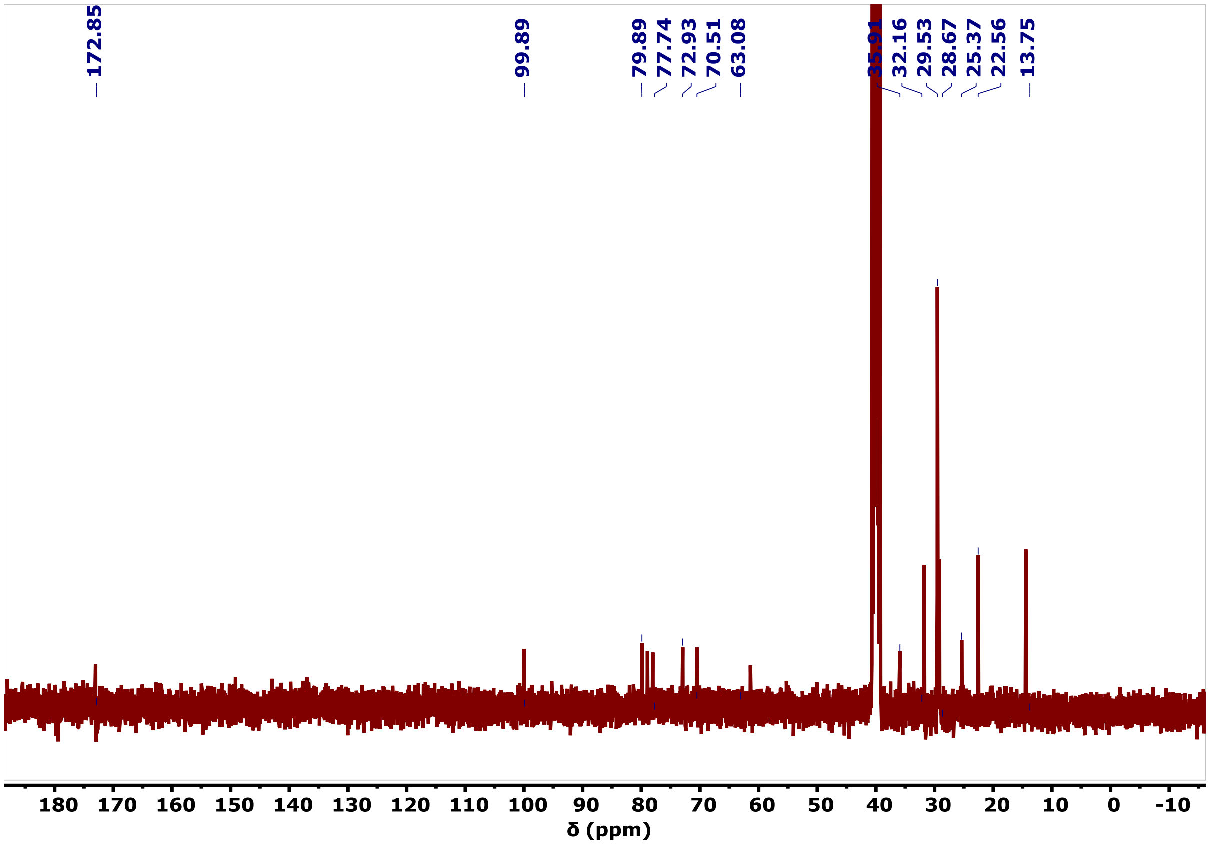

SI Fig. S4. ^{13}C NMR data of GLPA-C1.

^{13}C NMR (101 MHz, DMSO) δ 172.85, 99.89, 79.89, 77.74, 72.93, 70.51, 63.08, 35.91, 32.16, 29.53, 28.67, 25.37, 22.56, 13.75.

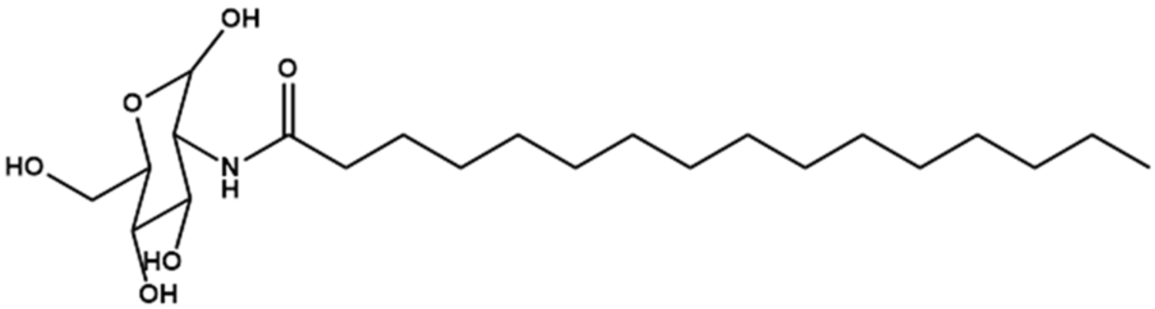

SI Fig. S5. Palmitic acid conjugated at the C2 position of Glucose **(GLPA-C2)**

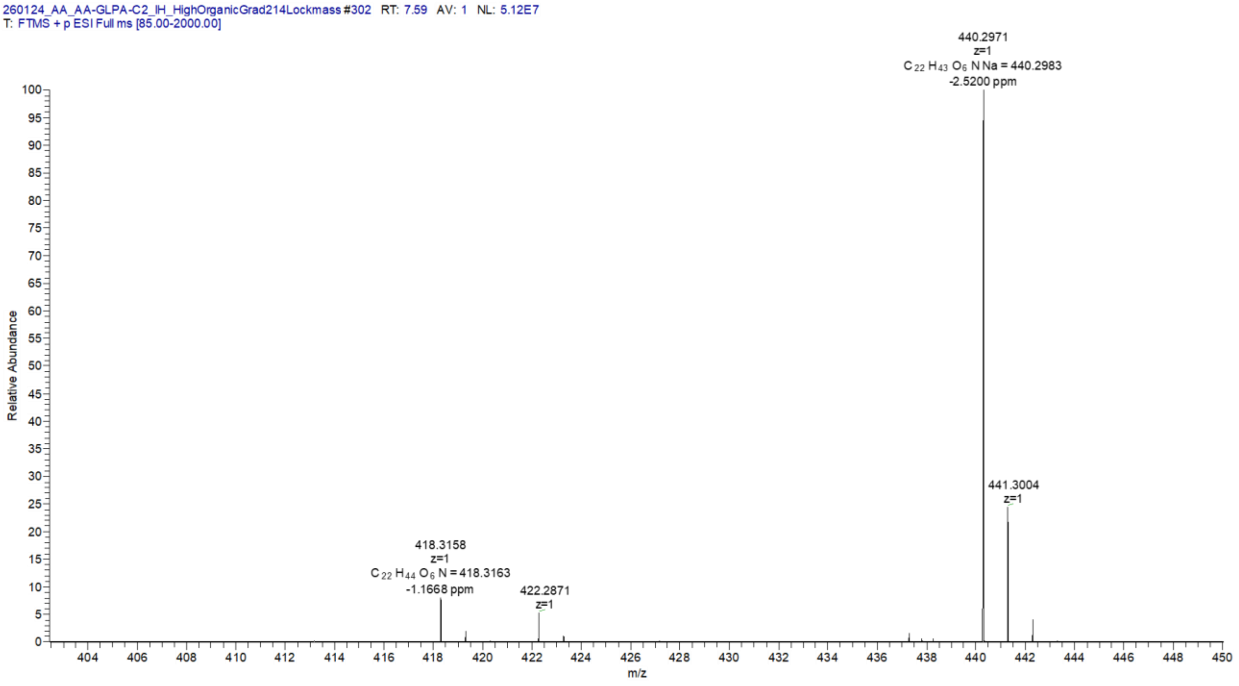

SI Fig. S6. Mass spectrometry data of GLPA-C2. $M_{(calculated)} = 417.59$ g mol^{-1}, $M_{(observed)} = 418.31$ g mol^{-1} (M+H), and 440.29 (M+Na).

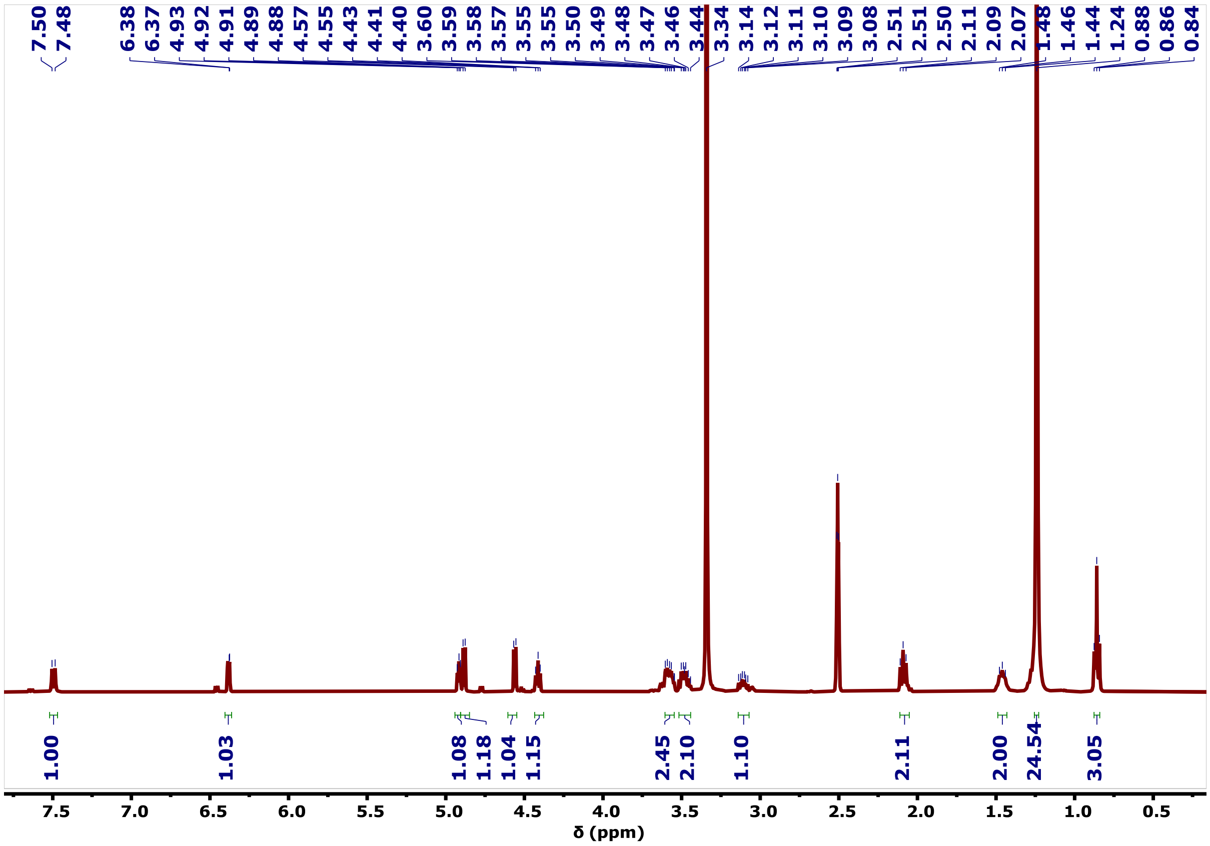

SI Fig. S7. ^{1}H NMR data of GLPA-C2.

^{1}H NMR (400 MHz, DMSO-d_6) δ 7.49 (d, J = 8.0 Hz, 1H), 6.38 (d, J = 1.1 Hz, 1H), 4.94 – 4.91 (m, 1H), 4.88 (d, J = 5.4 Hz, 1H), 4.56 (d, J = 5.5 Hz, 1H), 4.41 (t, J = 5.9 Hz, 1H), 3.58 (dd, J = 10.5, 4.9 Hz, 2H), 3.52 – 3.44 (m, 2H), 3.14 – 3.07 (m, 1H), 2.09 (t, J = 7.5 Hz, 2H), 1.47 (d, J = 7.3 Hz, 2H), 1.24 (s, 25H), 0.88 – 0.84 (m, 3H).

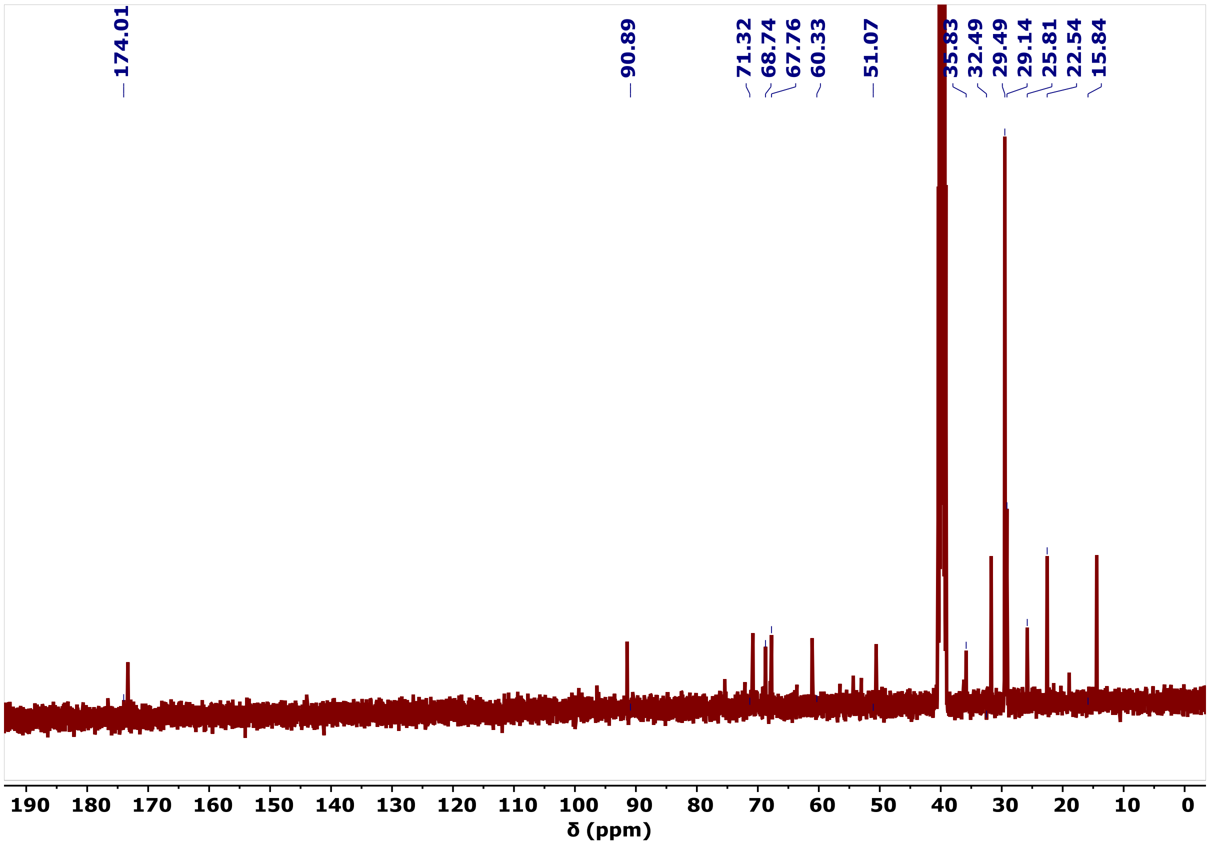

SI Fig. S8. ^{13}C NMR data of GLPA-C2.

^{13}C NMR (101 MHz, DMSO) δ 174.01, 90.89, 71.32, 68.74, 67.76, 60.33, 51.07, 35.83, 32.49, 29.49, 29.14, 25.81, 22.54, 15.84.

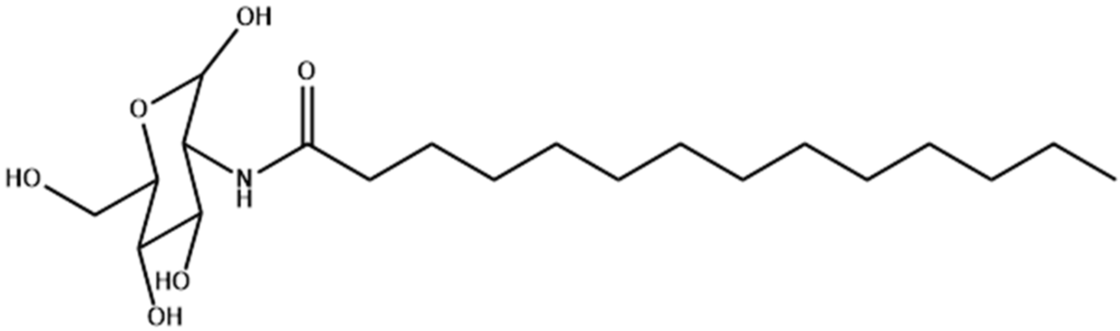

SI Fig. S9. Myristic acid conjugated at the C2 position of Glucose **(GLMY-C2)**.

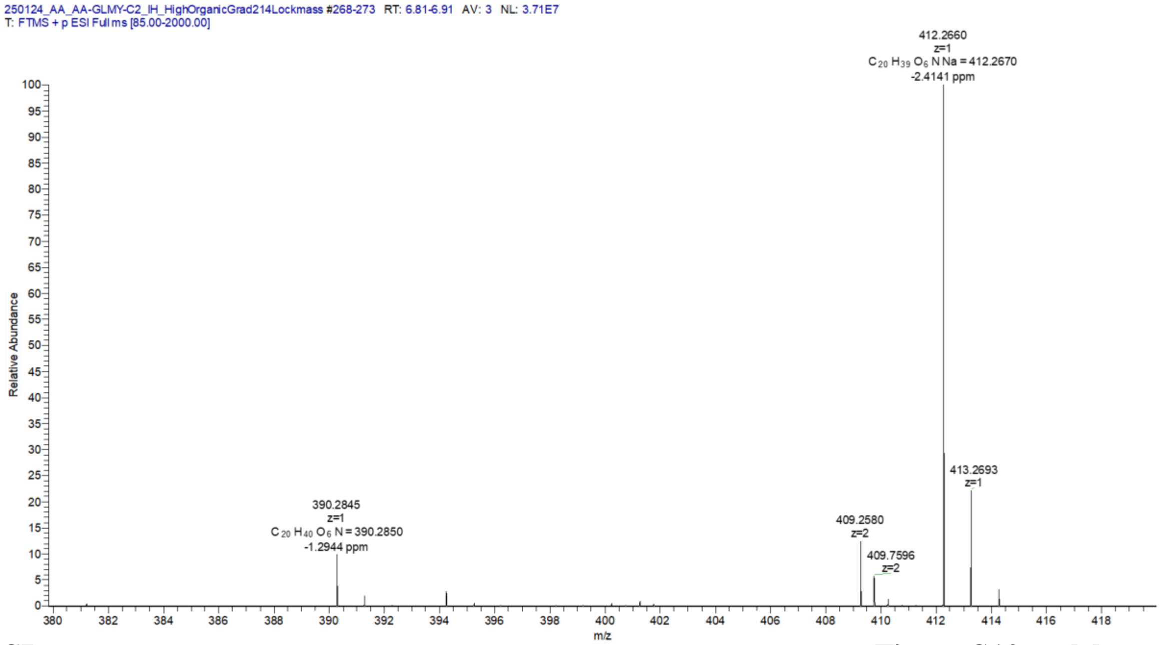

SI Fig. S10. Mass spectrometry data of GLMY-C2**.** $M_{(calculated)} = 389.53$, $M_{(observed)} = 390.28$ g mol^{-1} (M+H), and 412.26 g mol^{-1} (M+Na).

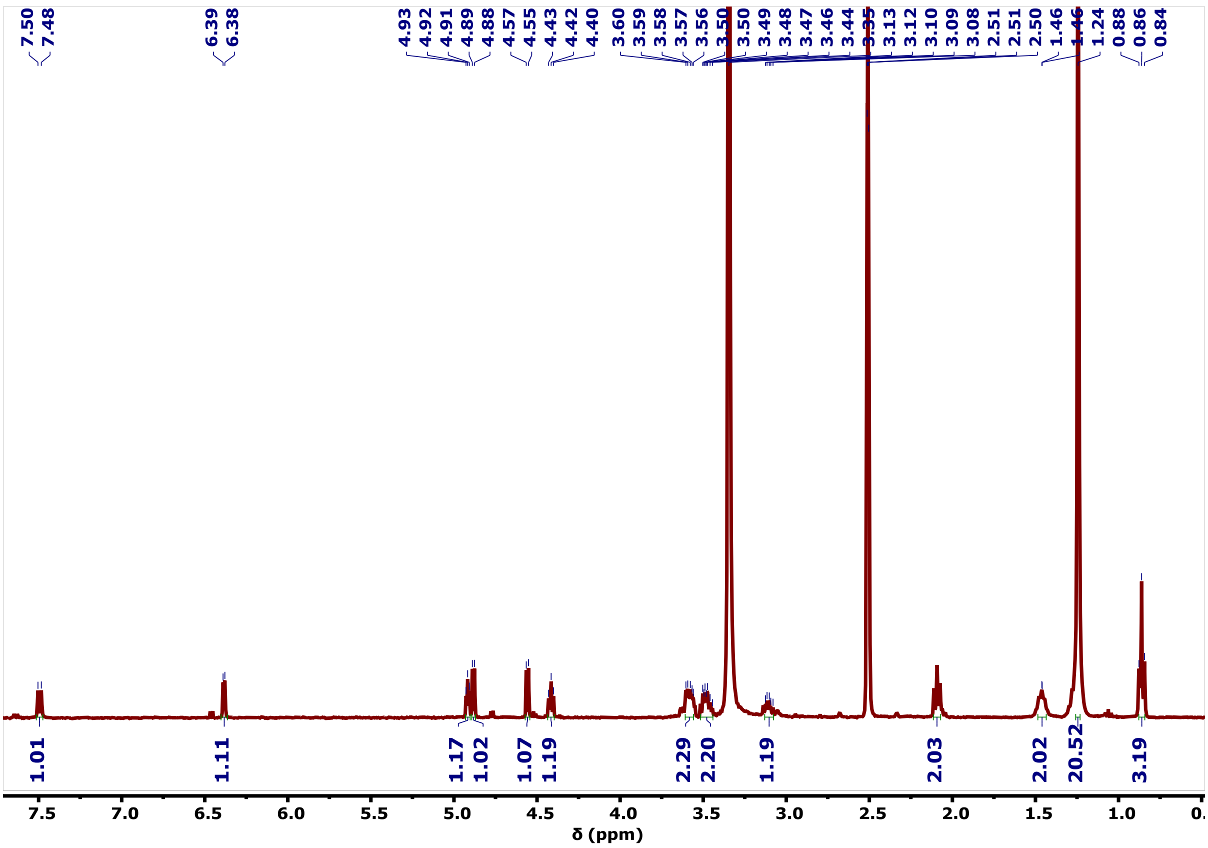

SI Fig. S11. ^{1}H NMR data of GLMY-C2**.**

^{1}H NMR (400 MHz, DMSO-d_6) δ 7.49 (d, J = 8.0 Hz, 1H), 6.38 (d, J = 4.4 Hz, 1H), 4.92 (t, J = 4.0 Hz, 1H), 4.88 (d, J = 5.4 Hz, 1H), 4.56 (d, J = 5.6 Hz, 1H), 4.42 (t, J = 5.9 Hz, 1H), 3.59 (dd, J = 10.5, 4.3 Hz, 2H), 3.52 – 3.44 (m, 2H), 3.10 (dt, J = 9.0, 4.6 Hz, 1H), 2.09 (t, J = 7.5 Hz, 2H), 1.49 – 1.44 (m, 2H), 1.24 (s, 20H), 0.88 – 0.84 (m, 3H).

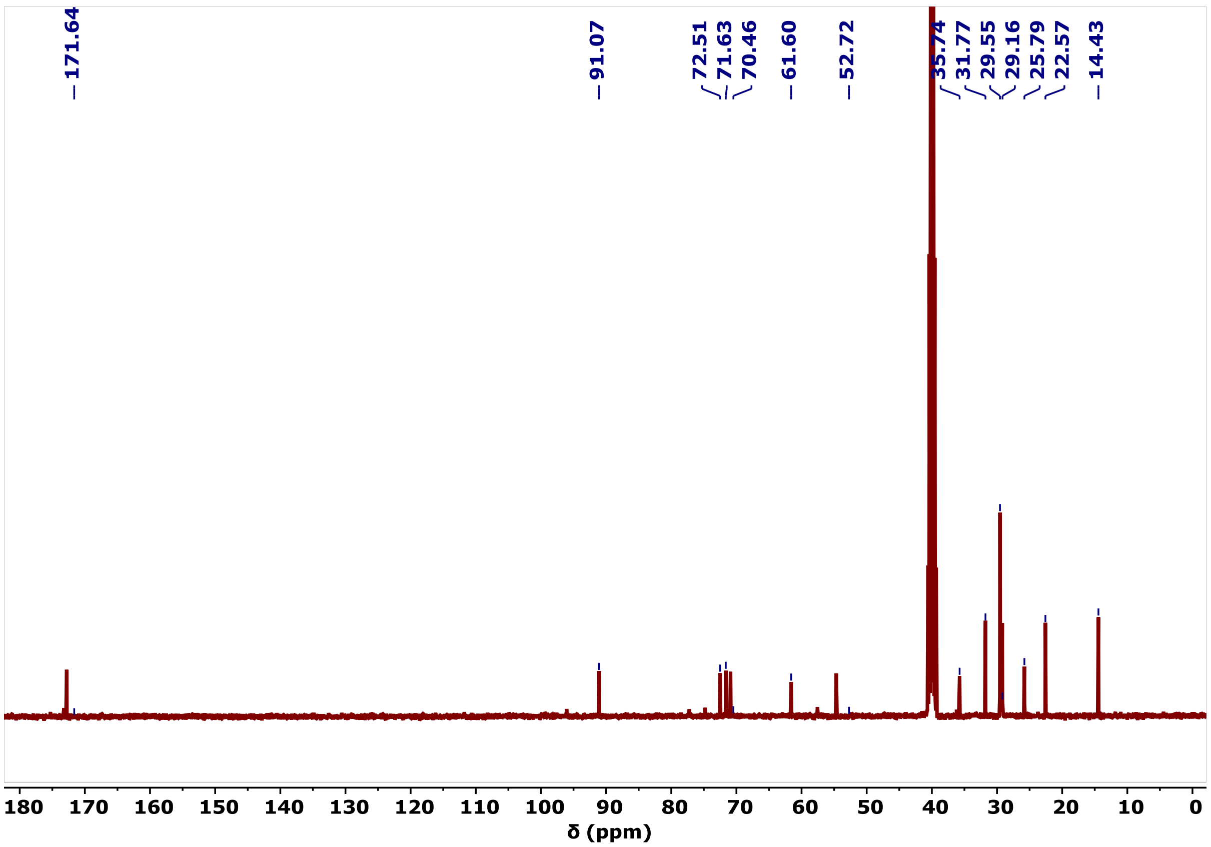

SI Fig. S12. ^{13}C NMR data of GLMY-C2.

^{13}C NMR (101 MHz, DMSO) δ 171.64, 91.07, 72.51, 71.63, 70.46, 61.60, 52.72, 35.74, 31.77, 29.55, 29.16, 25.79, 22.57, 14.43.

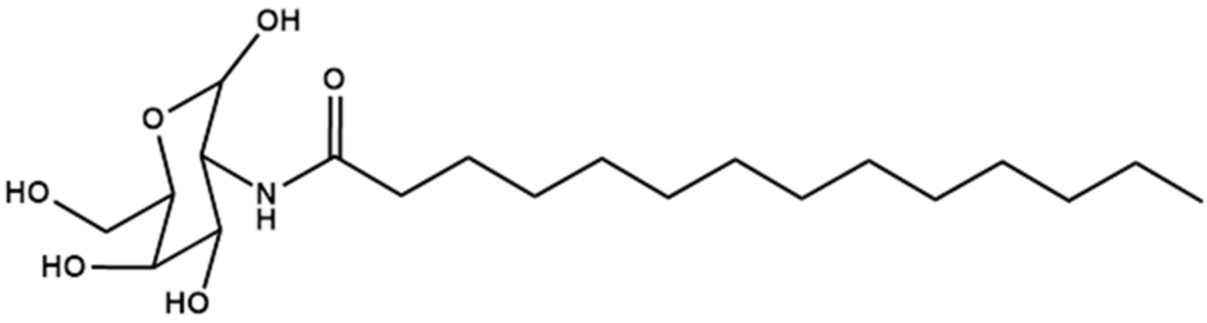

SI Fig. S13. Myristic acid conjugated at the C2 position of Galactose **(GALMY-C2)**.

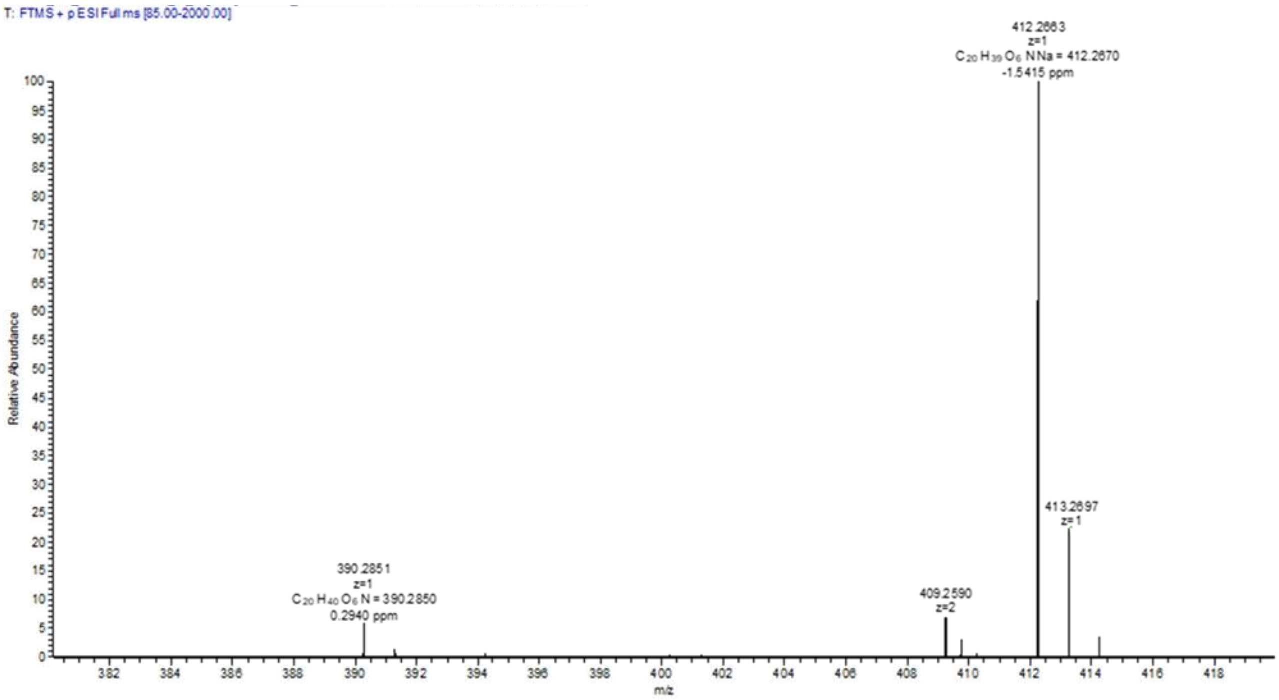

SI Fig.S14. Mass spectrometry data of GALMY-C2. M(calculated) = 389.53 g mol^{-1}, M(observed) = 390.28 g mol^{-1} (M+H), and 412.26 g mol^{-1} (M+Na).

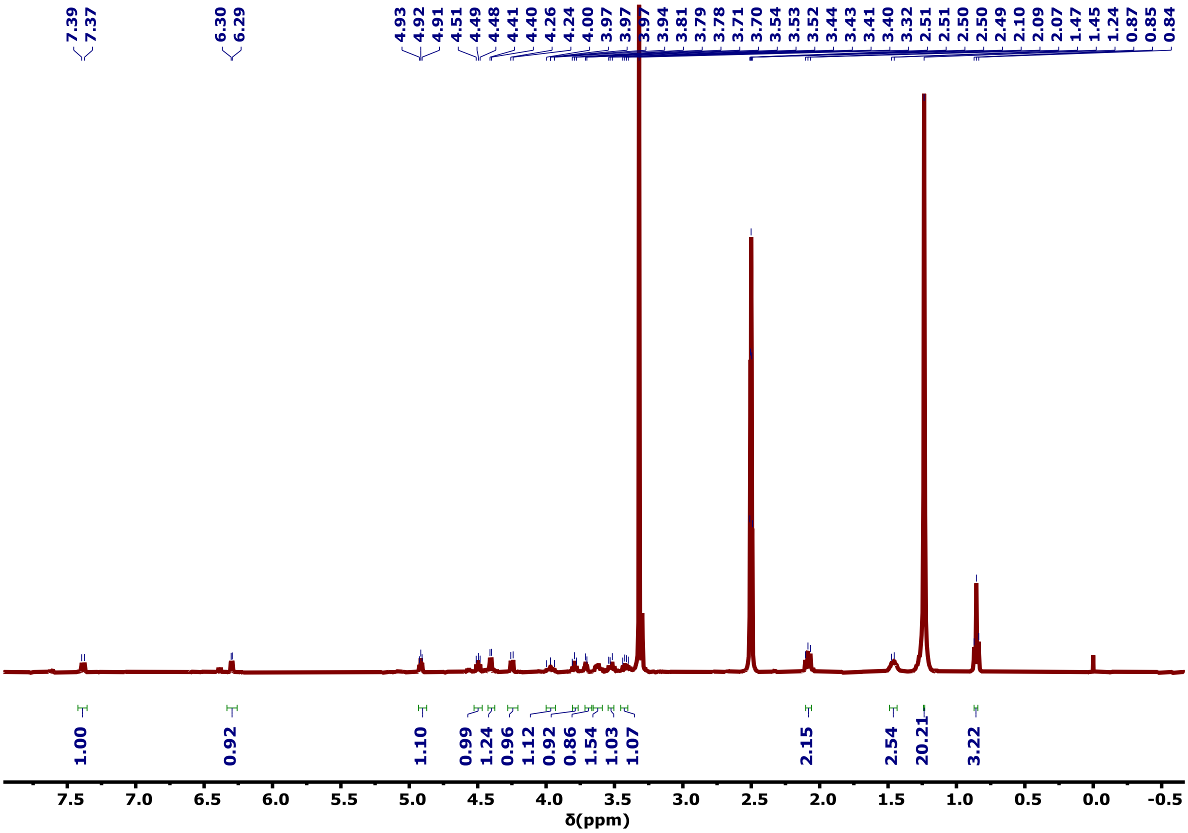

SI Fig. S15. ^{1}H NMR data of GALMY-C2.

1H NMR (400 MHz, DMSO-d6) δ 7.38 (d, J = 8.5 Hz, 1H), 6.30 (d, J = 3.3 Hz, 1H), 4.92 (t, J = 4.0 Hz, 1H), 4.53 – 4.47 (m, 1H), 4.40 (d, J = 4.3 Hz, 1H), 4.25 (d, J = 7.0 Hz, 1H), 4.00 – 3.93 (m, 1H), 3.78 (d, J = 6.3 Hz, 1H), 3.71 (d, J = 3.8 Hz, 1H), 3.66 – 3.59 (m, 2H), 3.55 – 3.50 (m, 1H), 3.45 – 3.40 (m, 1H), 2.08 (d, J = 7.5 Hz, 2H), 1.46 (d, J = 7.9 Hz, 3H), 1.24 (s, 20H), 0.86 (d, J = 6.7 Hz, 3H).

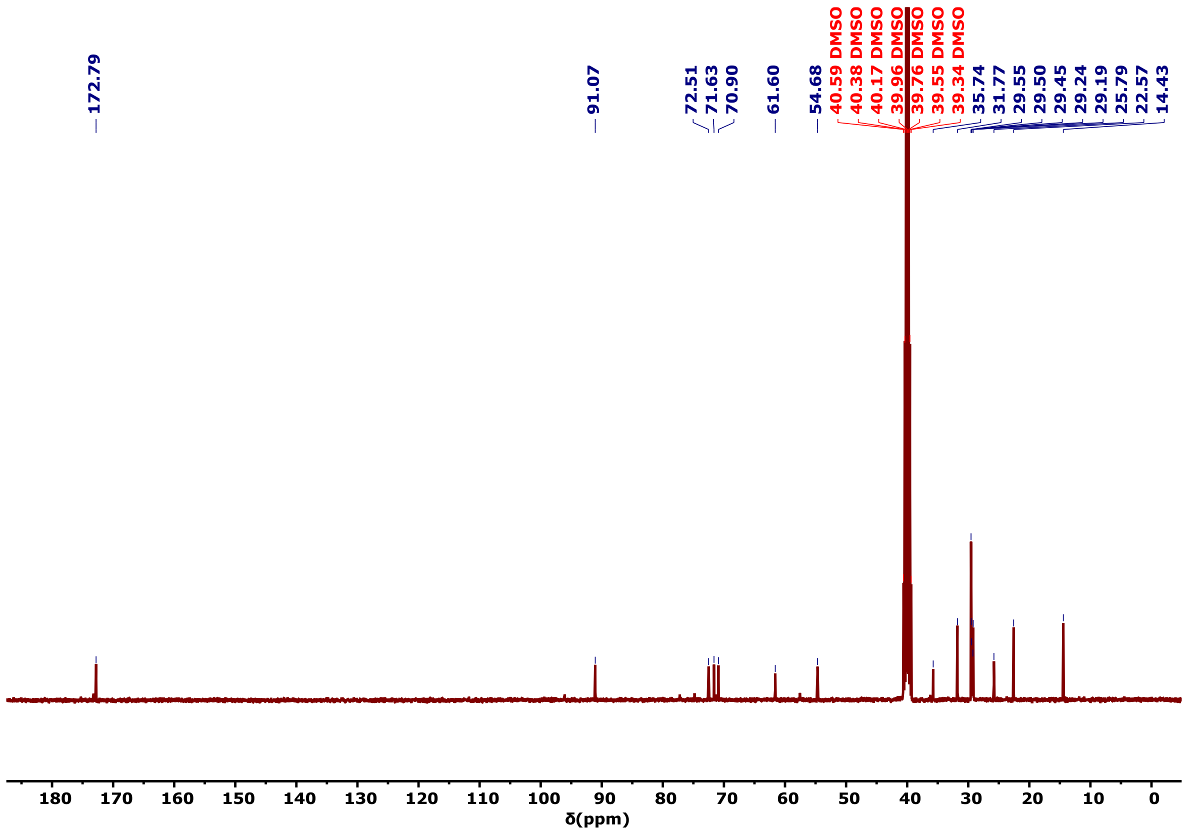

SI Fig. S16. ^{13}C NMR data of GALMY-C2**.**

13C NMR (101 MHz, DMSO) δ 172.79, 91.07, 72.51, 71.63, 70.90, 61.60, 54.68, 40.59, 40.38, 40.17, 39.96, 39.76, 39.55, 39.34, 35.74, 31.77, 29.55, 29.50, 29.45, 29.24, 29.19, 25.79, 22.57, 14.43.

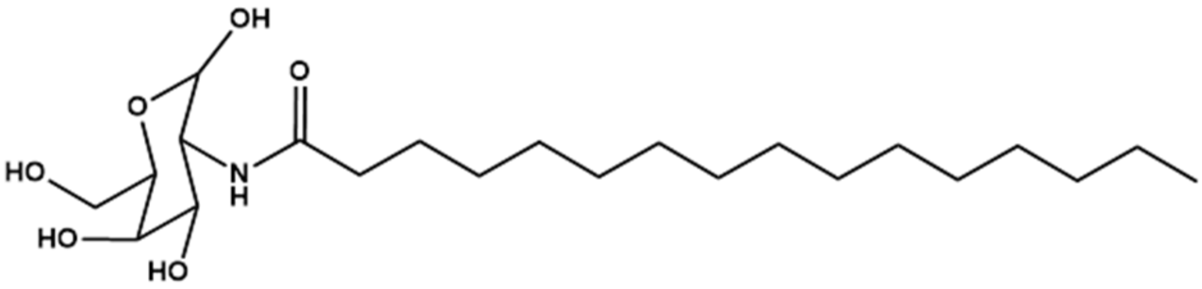

SI Fig. S17. Palmitic acid conjugated at the C2 position of Galactose **(GALPA-C2)**.

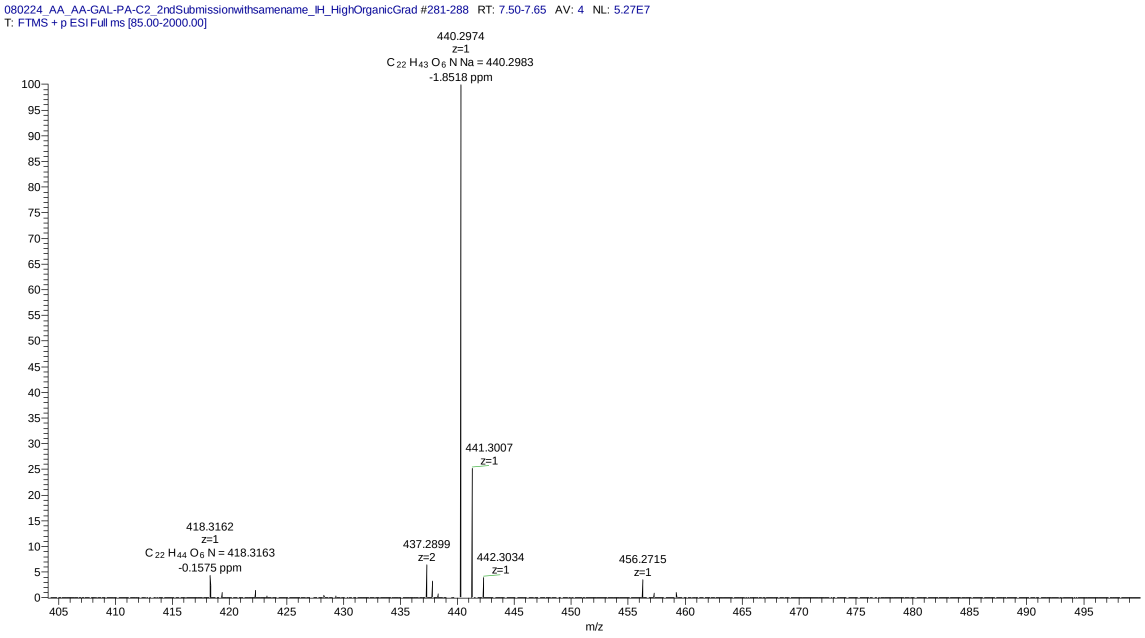

SI Fig. S18. Mass spectrometry data of GALPA-C2**.** $M_{(calculated)}$ = 417.59 g mol^{-1}, $M_{(observed)}$ = 418.31 g mol^{-1} (M+H), and 440.29 g mol^{-1} (M+Na).

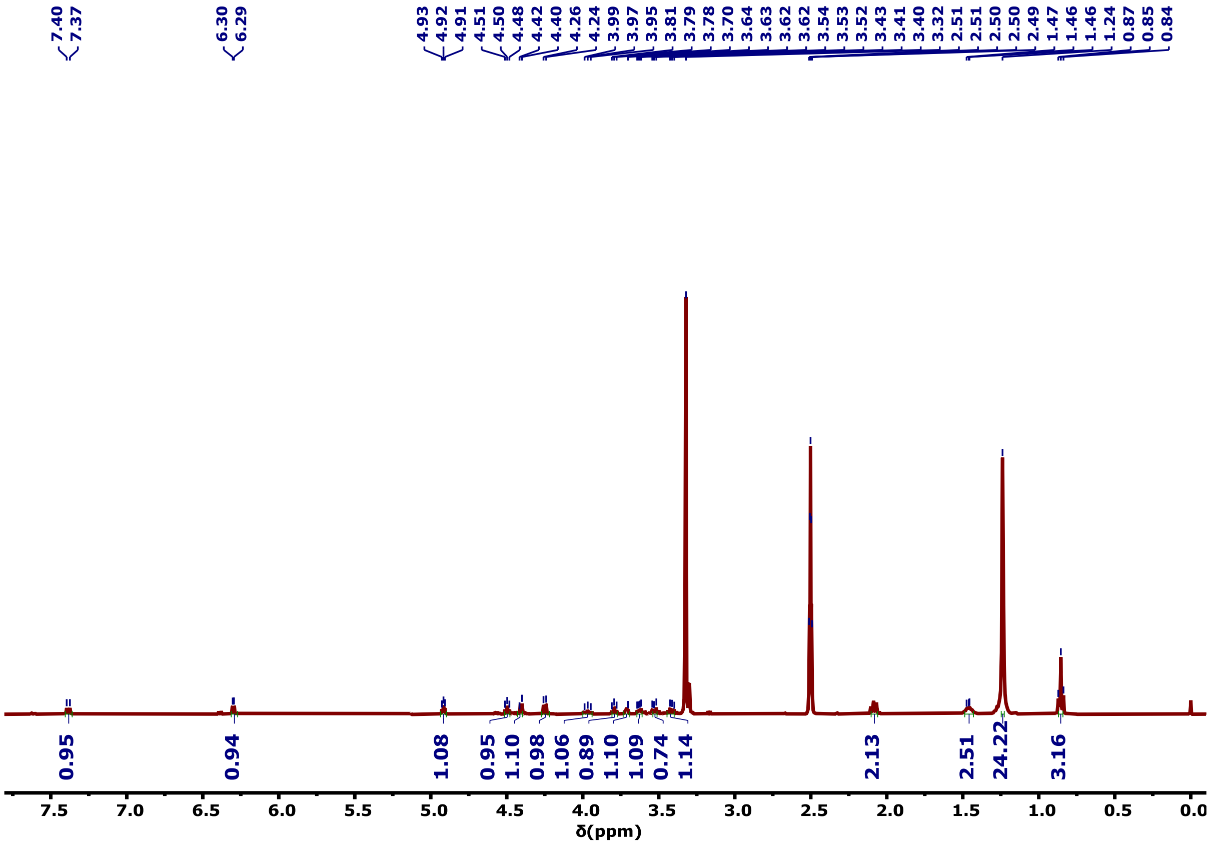

SI Fig. S19. ^{1}H NMR data of GALPA-C2.

^{1}H NMR (400 MHz, DMSO-d_6) δ 7.63 (d, J = 8.4 Hz, 1H), 7.40 (d, J = 8.5 Hz, 1H), 6.40 (d, J = 6.5 Hz, 1H), 6.32 (d, J = 4.4 Hz, 1H), 4.92 (d, J = 4.0 Hz, 1H), 4.60 (d, J = 5.6 Hz, 1H), 4.55 – 4.52 (m, 1H), 4.47 (d, J = 6.5 Hz, 1H), 4.43 (d, J = 2.2 Hz, 1H), 4.29 (d, J = 7.0 Hz, 1H), 3.99 – 3.94 (m, 1H), 3.80 (t, J = 6.3 Hz, 1H), 3.71 (s, 1H), 3.63 (s, 1H), 3.28 (t, J = 6.2 Hz, 1H), 2.07 (s, 2H), 1.47 (d, J = 7.2 Hz, 3H), 1.24 (s, 25H), 0.86 (s, 3H).

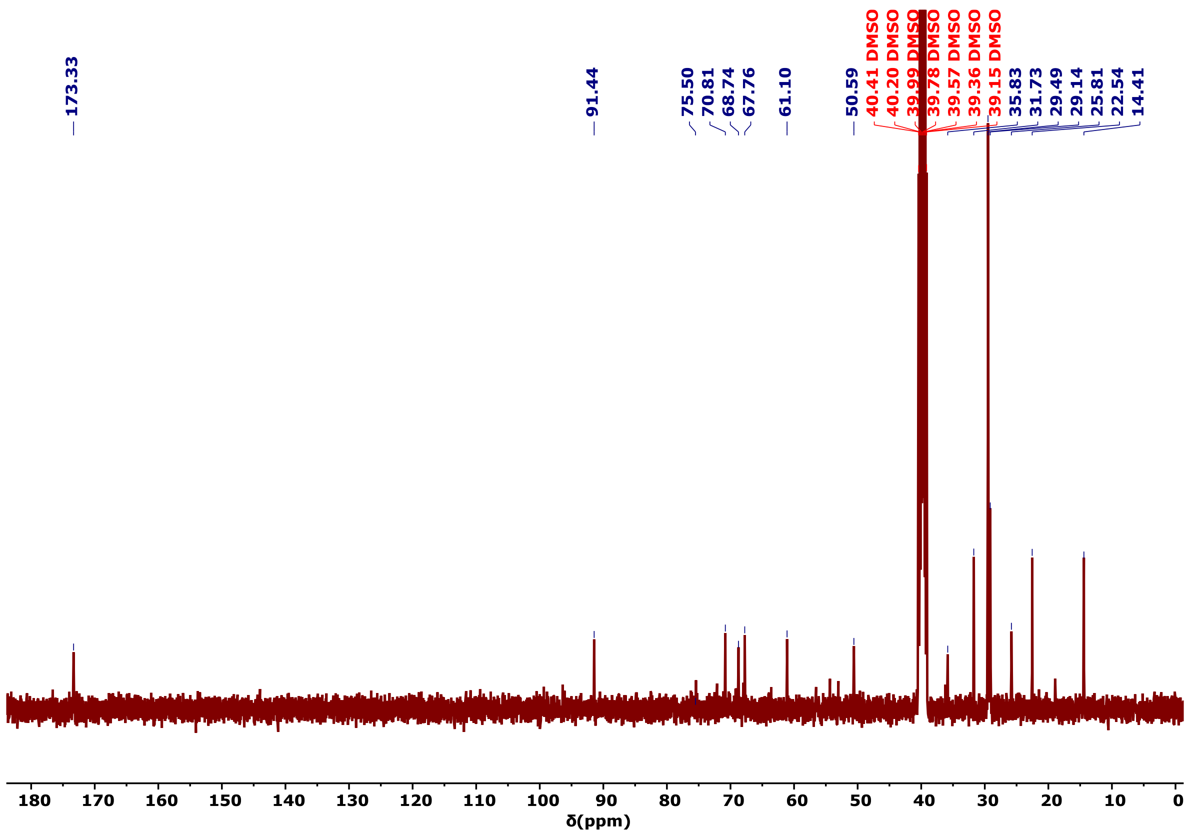

SI Fig. S20. ^{13}C NMR data of GALPA-C2**.**

13C NMR (101 MHz, DMSO) δ 173.33, 91.44, 75.50, 70.81, 68.74, 67.76, 61.10, 50.59, 40.41, 40.20, 39.99, 39.78, 39.57, 39.36, 39.15, 35.83, 31.73, 29.49, 29.14, 25.81, 22.54, 14.41.

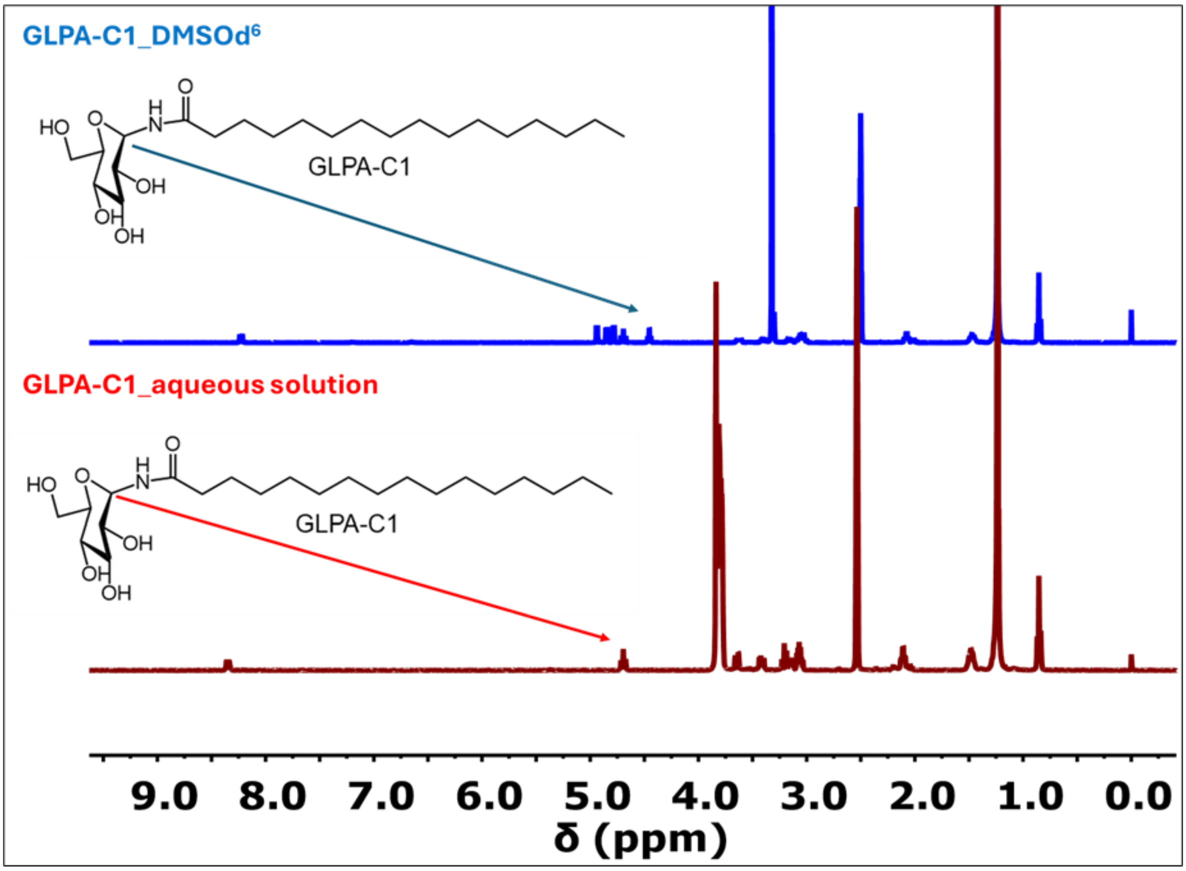

SI Fig. S21. ^{1}H NMR spectra of GLPA-C1 in DMSO-d_6 and water.

(a)

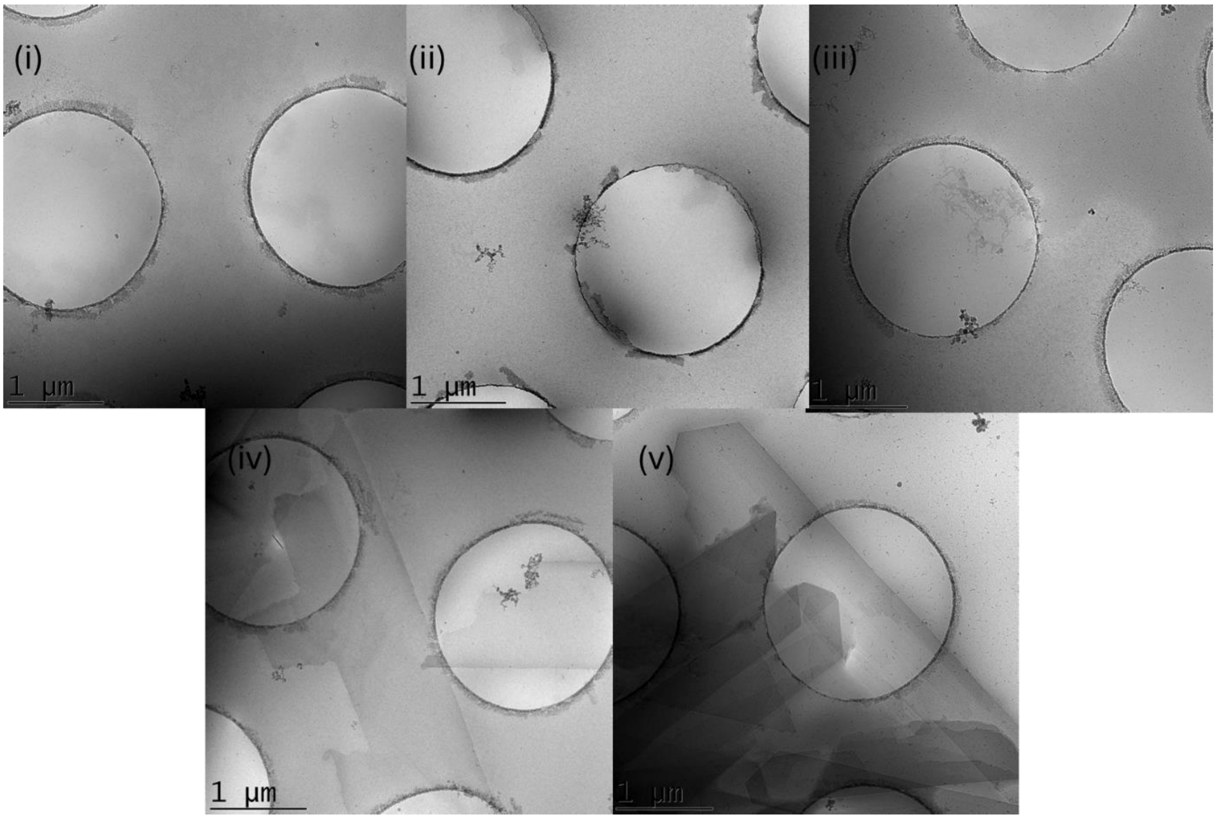

(b)

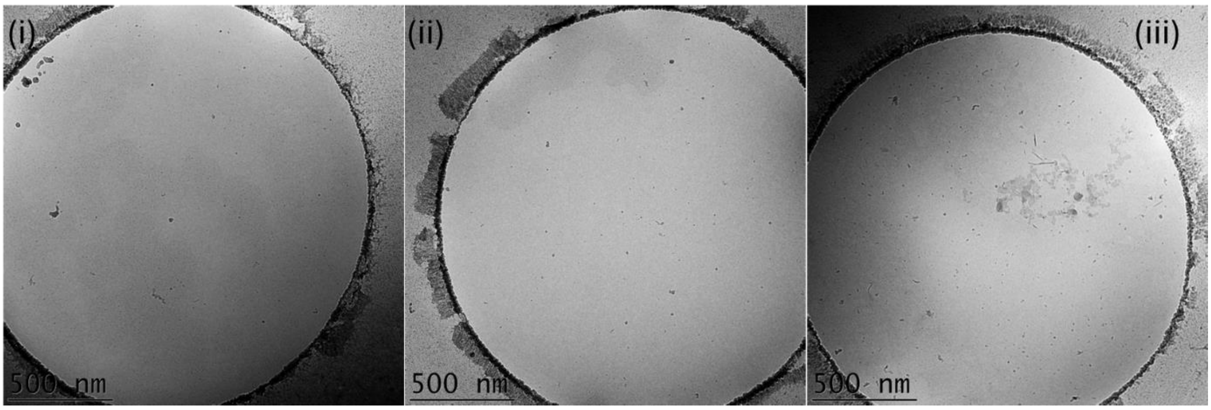

SI Fig. S22. (a) Additional cryo-TEM images for 0.1 wt% solutions in 10 wt% methanol/90 wt% water. (i) GLPA-C1, (ii) GLPA-C2, (iii) GLMY-C2, (iv) GALMY-C2, (v) GALPA-C2. (b) Enhanced contrast images for (i) GLPA-C1, (ii) GLPA-C2 and (iii) GLMY-C2.

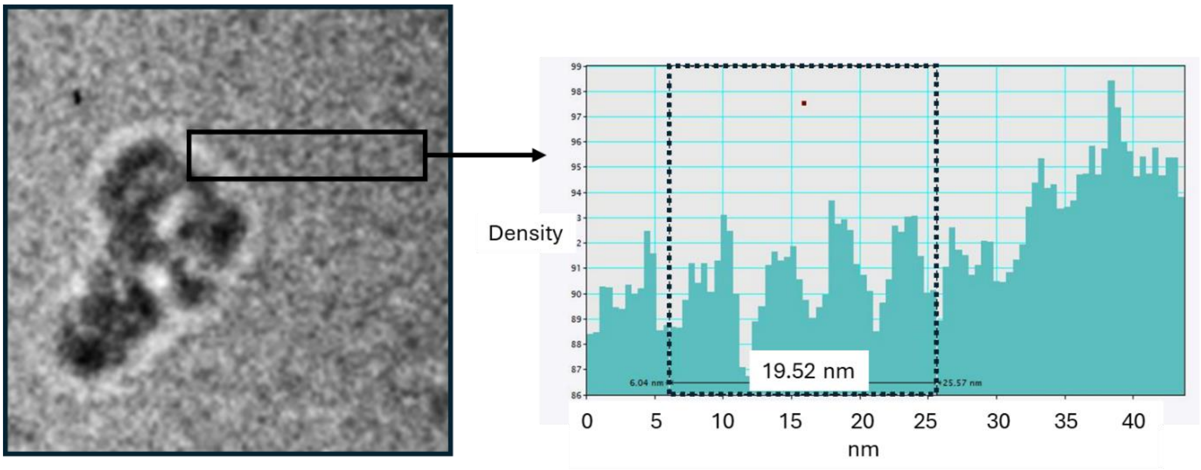

SI Fig.S23. Example of region of cryo-TEM image from GLPA-C2 showing fringes from small multilamellar stack along with cross-section analysis to estimate periodicity, 4.8 nm.

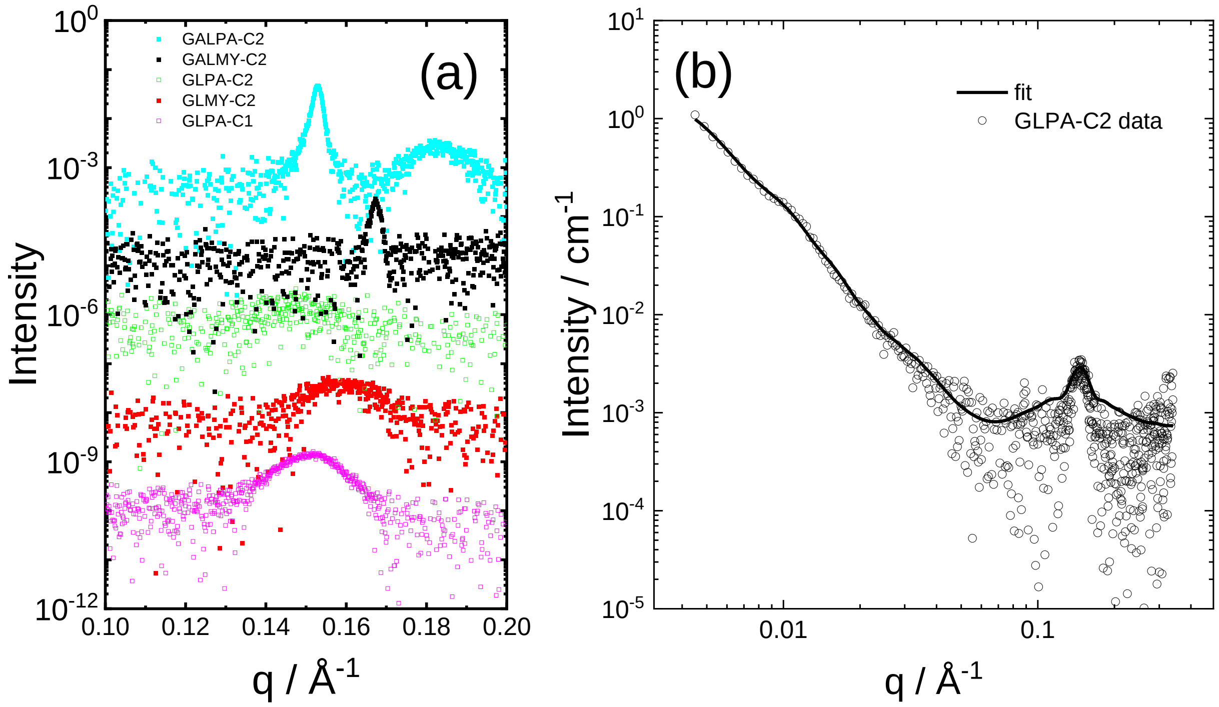

SI Fig. S24. (a) SAXS data (same data as Fig.5) on expanded linear *q* scale. Data are offset for ease of visualization, (b) Fit of data for GLPA-C2 (double log scale, fit parameters in SI Table S1). For ease of visualization, only every 3rd data point is shown.

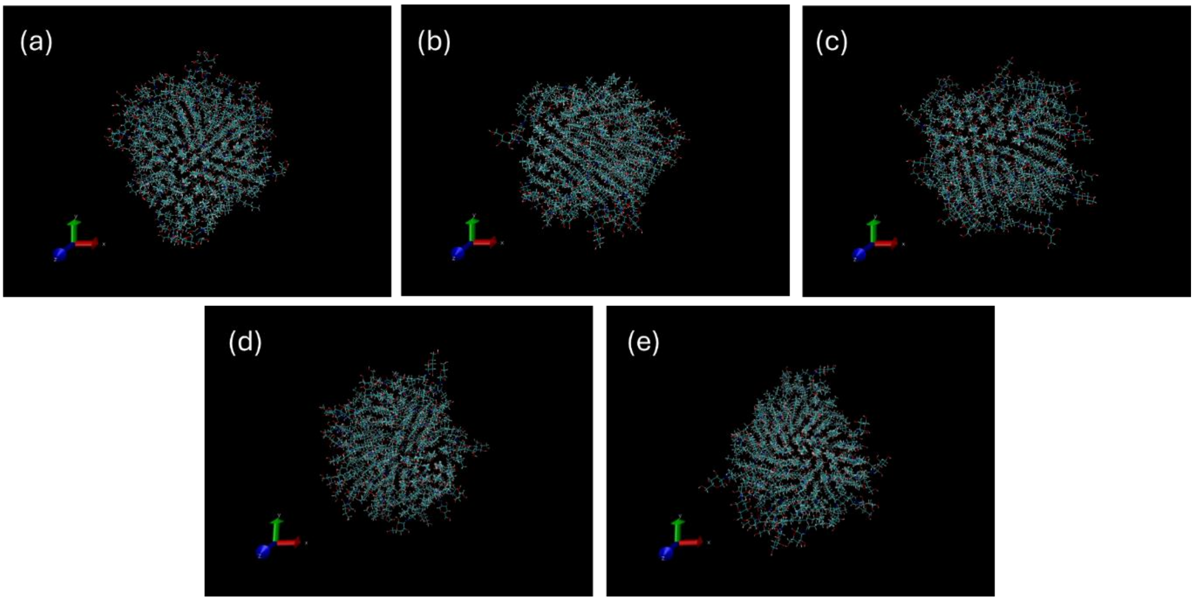

SI Fig. S25. Images of in-plane ordering from final frames of MD simulations for (a) GLPA-C1, (b) GLPA-C2, (c) GLMY-C2, (d) GALMY-C2, (e) GALPA-C2.

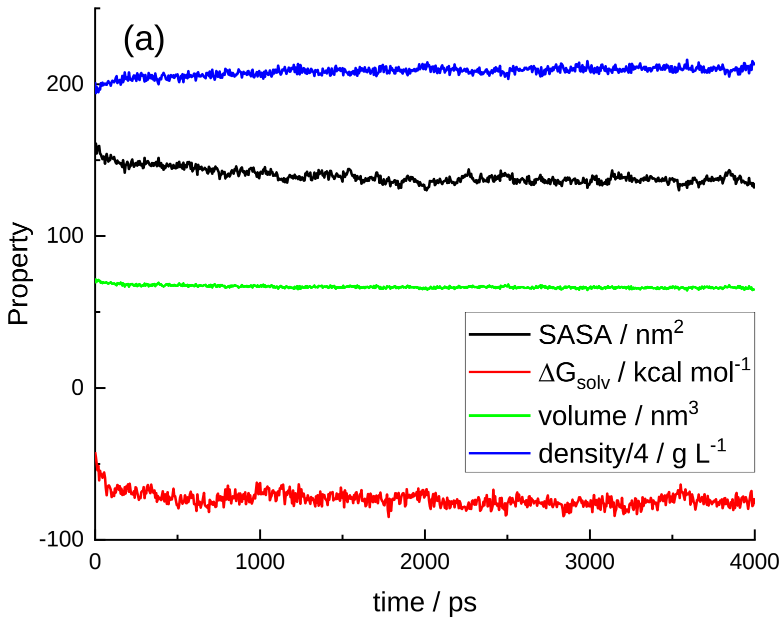

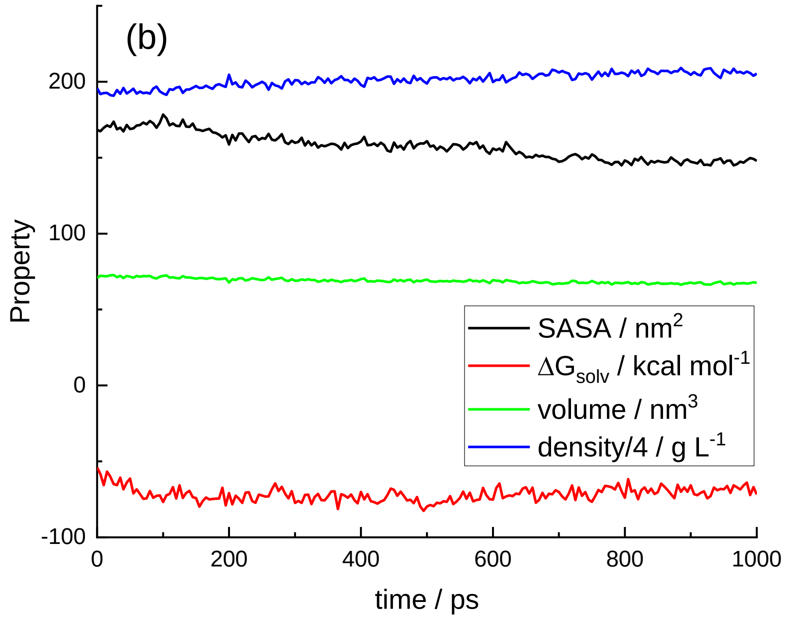
(b)
200
100
0
-100
Property
0
200
400
600
800
1000
time / ps
SASA / nm2
ΔGsolv / kcal mol-1
volume / nm3
density/4 / g L-1

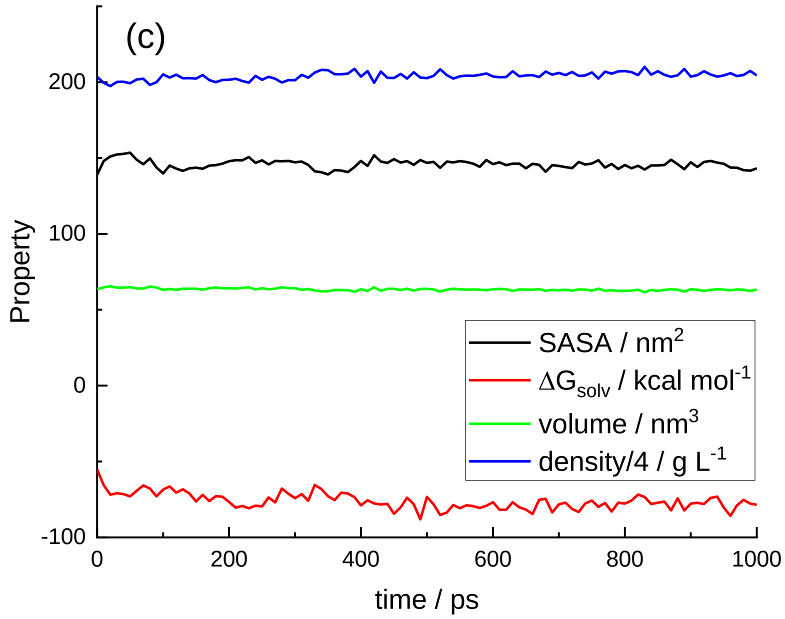
(c)
200
100
0
-100
Property
0
200
400
600
800
1000
time / ps
SASA / nm2
ΔGsolv / kcal mol-1
volume / nm3
density/4 / g L-1

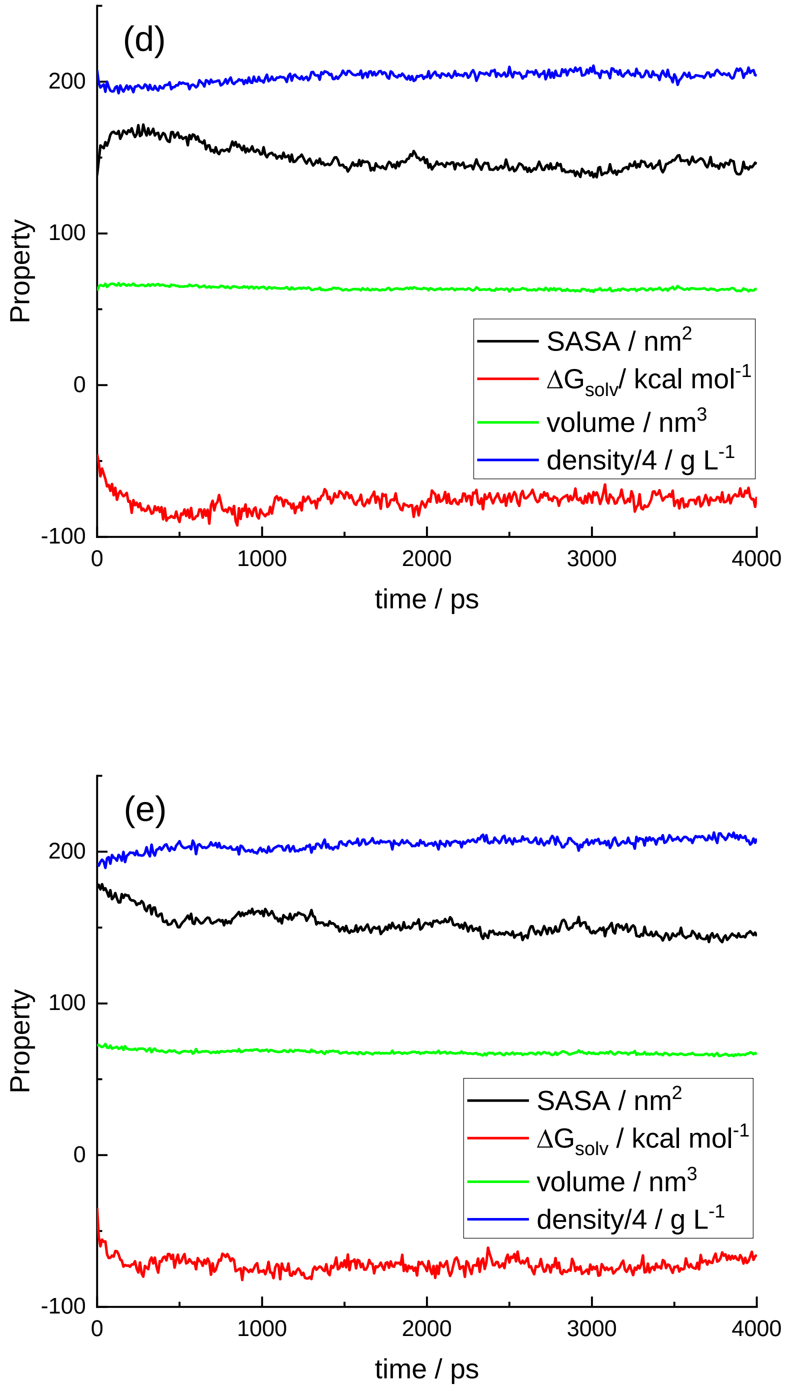

SI Fig. S26. SASA-related properties from MD simulations for (a) GLPA-C1, (b) GLPA-C2, (c) GLMY-C2, (d) GALMY-C2, (e) GALPA-C2.

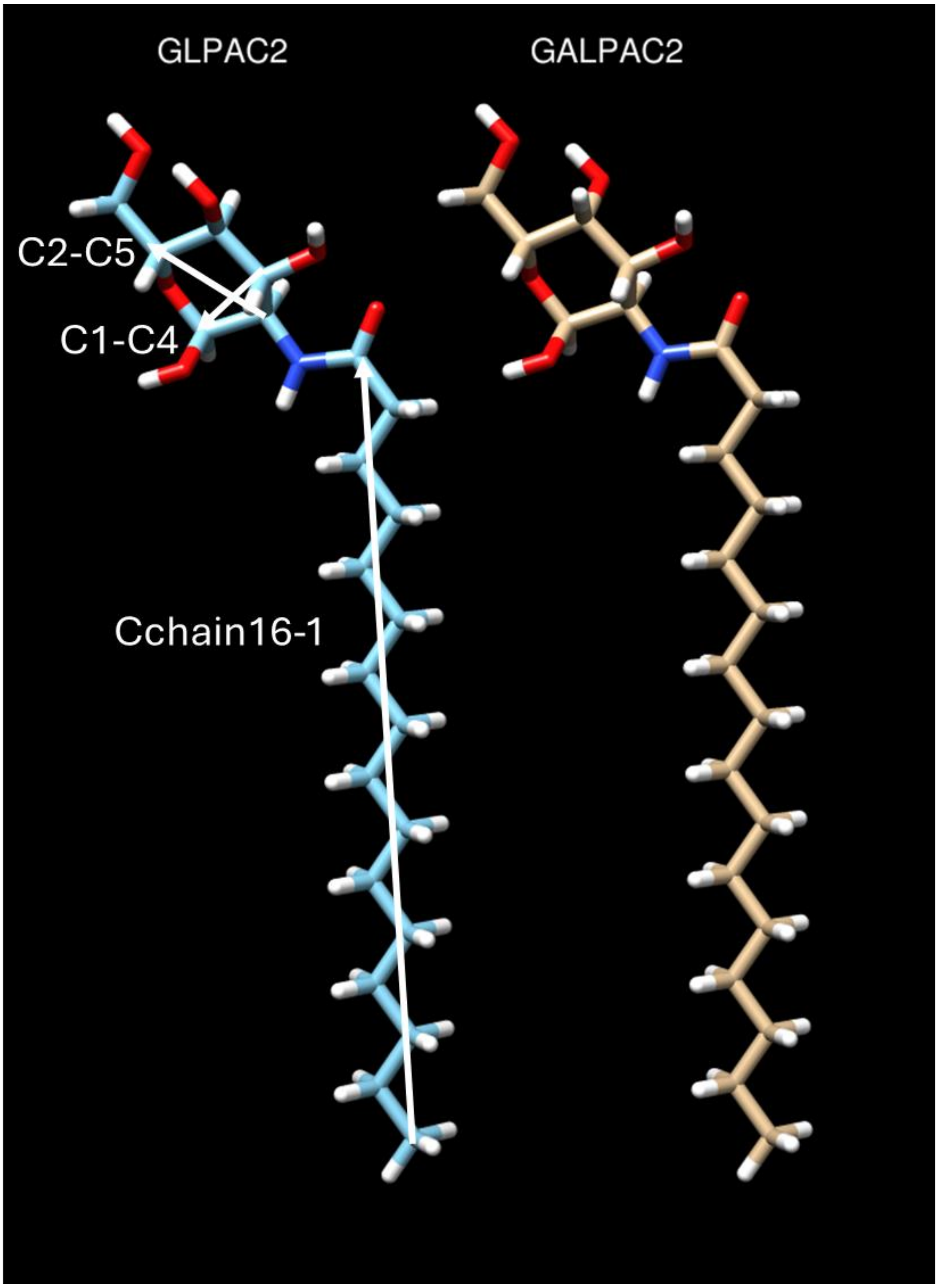

SI Fig.S27. Molecular structures of GLPA-C2 and GALPA-C2 and definitions of vectors for MD angle distribution studies.

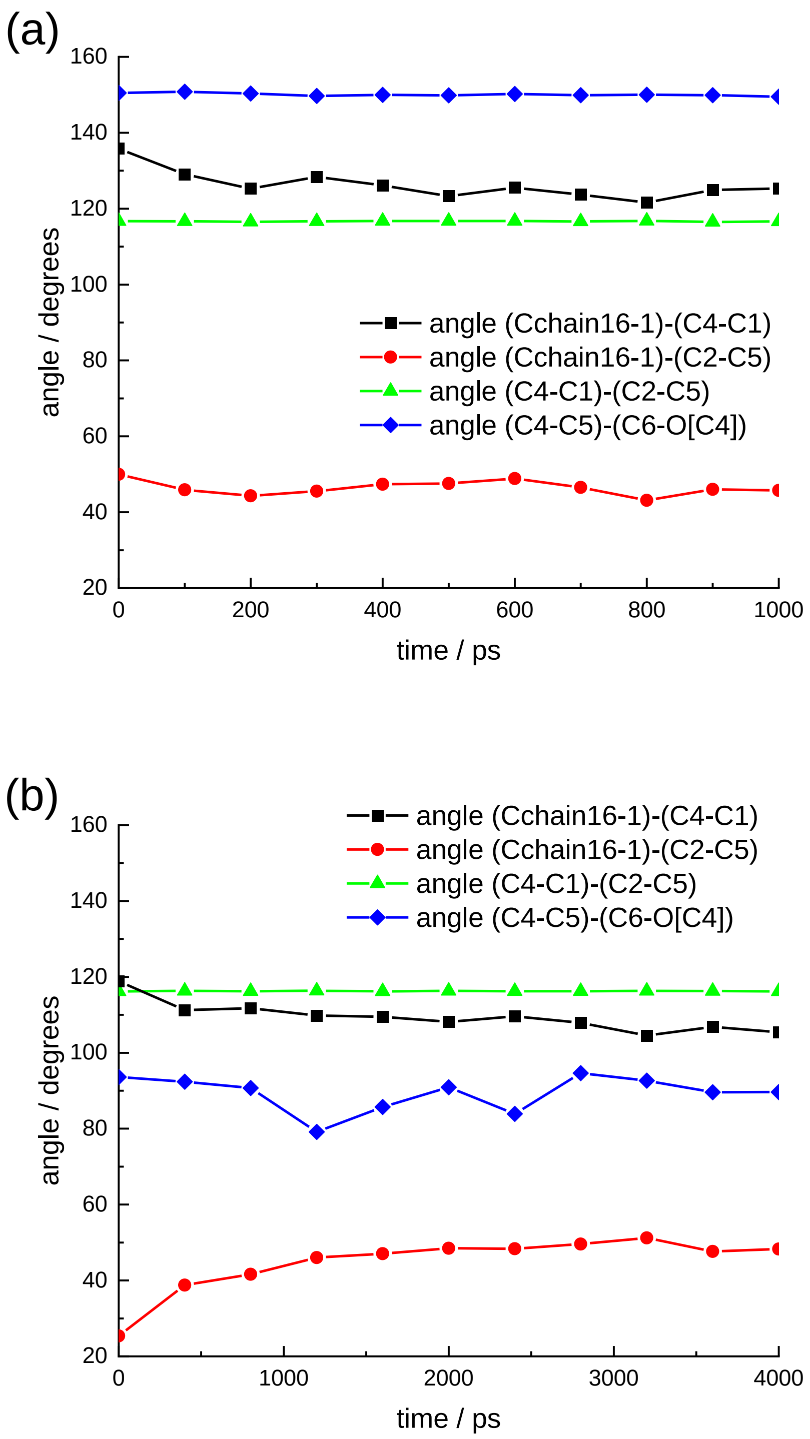

SI Fig.S28. Angles associated with packing of lipid chains/headgroups and vectors within sugar rings from MD simulations for (a) GLPA-C2, (b) GALPA-C2. See Fig.1b and SI Fig.S25 for atom labelling.

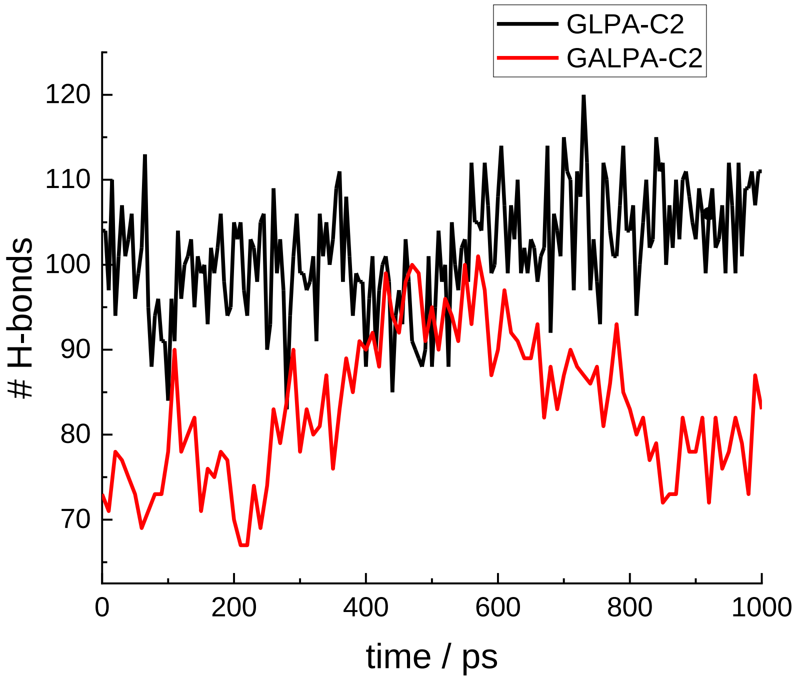

SI Fig.S29. Time evolution of numbers of hydrogen bonds (within the whole system) for GLPA-C2 and GALPA-C2.

Table S1. Parameters extracted from the fitting of the SAXS data SI Fig.S24b for GLPA-C2.[a]

	**GLPA-C2 0.1% in 10% methanol/90% water**
$r_H \pm \Delta r_H$ [Å]	31.4 ± 6.7
ρ_{H}	2.07×10^{-8}
σ_{H} [Å][b]	5.0
ρ_{C}	-2.01×10^{-8}
σ_{C} [Å][b]	5.0
D [Å]	1093
N	5.6
d [Å]	42.8
η[b]	0.1
ν	11.7
C	1.1×10^{-4}

[a] Data fitted using the software SASfit.[1-2]

[b] Fixed parameter

Key: Gaussian bilayer: layer thickness r_H (Gaussian polydispersity Δr_H), scattering contrast of outer (headgroup) layers ρ_{H}, and core (lipid chain) layer ρ_{C}, Gaussian widths σ_{C} and σ_{H} of core and headgroup layers respectively, D diameter (width) of layer system (when $D >> t$ as here, it acts as a scaling parameter for the form factor). **Modified Caillé Lamellar Structure Factor:** number of layers N, layer period d, Caillé parameter η, diffuse scattering term (number of uncorrelated bilayers) ν. **Background:** constant background, C.

References

(1) Bressler, I.; Kohlbrecher, J.; Thünemann, A. F., SASfit: a tool for small-angle scattering data analysis using a library of analytical expressions. *Journal of Applied Crystallography* **2015,** *48,* 1587-1598.

(2) Kohlbrecher, J.; Bressler, I., Updates in SASfit for fitting analytical expressions and numerical models to small-angle scattering patterns. *Journal of Applied Crystallography* **2022,** *55,* 1677-1688.
